# Three-Week-Old Rabbit Ventricular Cardiomyocytes as a Novel System to Study Cardiac Excitation and EC Coupling

**DOI:** 10.3389/fphys.2021.672360

**Published:** 2021-11-18

**Authors:** Anatoli Y. Kabakov, Elif Sengun, Yichun Lu, Karim Roder, Peter Bronk, Brett Baggett, Nilüfer N. Turan, Karni S. Moshal, Gideon Koren

**Affiliations:** ^1^Department of Medicine, Division of Cardiology, Cardiovascular Research Center, Rhode Island Hospital, The Warren Alpert Medical School of Brown University, Providence, RI, United States; ^2^Department of Pharmacology, Institute of Graduate Studies in Health Sciences, Istanbul University, Istanbul, Türkiye

**Keywords:** cardiac ventricular myocytes, cultured, rabbit, EC coupling, patch clamp, cardiac excitation, drug discovery

## Abstract

Cardiac arrhythmias significantly contribute to cardiovascular morbidity and mortality. The rabbit heart serves as an accepted model system for studying cardiac cell excitation and arrhythmogenicity. Accordingly, primary cultures of adult rabbit ventricular cardiomyocytes serve as a preferable model to study molecular mechanisms of human cardiac excitation. However, the use of adult rabbit cardiomyocytes is often regarded as excessively costly. Therefore, we developed and characterized a novel low-cost rabbit cardiomyocyte model, namely, 3-week-old ventricular cardiomyocytes (3wRbCMs). Ventricular myocytes were isolated from whole ventricles of 3-week-old New Zealand White rabbits of both sexes by standard enzymatic techniques. Using wheat germ agglutinin, we found a clear T-tubule structure in acutely isolated 3wRbCMs. Cells were adenovirally infected (multiplicity of infection of 10) to express Green Fluorescent Protein (GFP) and cultured for 48 h. The cells showed action potential duration (APD90 = 253 ± 24 ms) and calcium transients similar to adult rabbit cardiomyocytes. Freshly isolated and 48-h-old-cultured cells expressed critical ion channel proteins: calcium voltage-gated channel subunit alpha1 C (Ca_v_α1c), sodium voltage-gated channel alpha subunit 5 (Nav1.5), potassium voltage-gated channel subfamily D member 3 (Kv4.3), and subfamily A member 4 (Kv1.4), and also subfamily H member 2 (RERG. Kv11.1), KvLQT1 (K7.1) protein and inward-rectifier potassium channel (Kir2.1). The cells displayed an appropriate electrophysiological phenotype, including fast sodium current (*I*_Na_), transient outward potassium current (*I*_to_), L-type calcium channel peak current (*I*_Ca,L_), rapid and slow components of the delayed rectifier potassium current (*I*_Kr_ and *I*_Ks_), and inward rectifier (*I*_K1_). Although expression of the channel proteins and some currents decreased during the 48 h of culturing, we conclude that 3wRbCMs are a new, low-cost alternative to the adult-rabbit-cardiomyocytes system, which allows the investigation of molecular mechanisms of cardiac excitation on morphological, biochemical, genetic, physiological, and biophysical levels.

## Introduction

Cardiac arrhythmias significantly contribute to cardiovascular morbidity and mortality (Stewart et al., 2002; Schnabel et al., 2015; Grandi et al., 2017) and are associated with cardiac remodeling characterized by changes in ion channel function, calcium homeostasis, and cell structure (Schotten et al., 2011; Chiamvimonvat et al., 2017; Grandi et al., 2017). To study cardiac electrophysiology, calcium homeostasis, biomechanics, cell morphology, signal transduction, protein, or gene expression, isolated cardiomyocytes have been the preferred choice as they are more physiologically relevant than immortalized cardiac cell lines such as murine HL-1 cells, which are derived from the AT-1 atrial tumor cell lineage and do not recapitulate ventricular cells in culture (Peter et al., 2016). Over the decades, many techniques for cardiomyocyte isolation have been published, e.g., isolations from adult or neonatal cardiomyocytes from mice (Ehler et al., 2013; Ackers-Johnson et al., 2016), rats (Vandergriff et al., 2015; Nippert et al., 2017), rabbits (Ziv et al., 2009), etc. For reviews, see Camacho et al. (2016); Quinn and Kohl (2016), and/or Clauss et al. (2019).

Although guinea pig left ventricular myocytes have an action potential (AP) duration (APD90) (Beserra et al., 2020; Al-Owais et al., 2021) similar to rabbit APD90, they lack transient outward potassium current (*I*_to_) (Hoppe et al., 1999), which is critical for early phase 1 repolarization during the cardiac AP (Varro et al., 1993; Dixon et al., 1996). In larger mammals, *I*_to_ establishes the membrane potential for Ca^2+^ entry and influences APD, and changes in *I*_to_ may underlie ventricular tachycardia (Choi et al., 2018).

In recent years, significant progress has been made in the development of induced pluripotent stem cells (iPSC) and the generation of iPSC-derived cardiomyocytes (iPSC-CM) with respect to quality, differentiation efficiency, or maturity (Bedada et al., 2016; Machiraju and Greenway, 2019). iPSC-CMs are relatively more immature and similar to fetal rather than adult cardiomyocytes. These cells can serve as an excellent model to study human mutations and relevant therapeutic approaches. However, compared to rod-shaped adult cardiomyocytes with an organized sarcomere and T-tubule structure, iPSC-CMs, similar to embryonic cardiomyocytes, are generally smaller, with disorganized sarcomeres and lack of T-tubules and key currents such as Kir2.1 and Kv7.1. Thus, due to the immaturity of neonatal cardiomyocytes or iPSC-CMs, adult cardiomyocytes are still preferred in the study of excitation-contraction coupling and cellular electrophysiology.

In contrast to rodents, the rabbit heart is more similar to the human heart with respect to heart rate, AP shape, and EC coupling. Therefore, the rabbit heart serves as a well-accepted model system for the human heart in electrophysiology and structure, and primary cultures of adult rabbit ventricular cardiomyocytes are one of the preferable models to study molecular mechanisms similar to those of human ventricular cardiomyocytes (Gemmell et al., 2016; Vinogradova et al., 2018; Shiomi, 2019a,b). However, the use of adult rabbit cardiomyocytes is often regarded as rather expensive and hinders the transition of a researcher from rodent to rabbit cardiomyocytes. Previously, we have shown that neonatal rabbit cardiomyoytes (NRbCMs) have several advantages over adult cardiomyocytes including easier cell isolation, culture, and amenability to liposome-mediated transfection. We utilized NRbCMs for genetic manipulations, biochemical assays, measuring of membrane potential waveform, and calcium transients by optical methods (Moshal et al., 2014; Roder et al., 2014, 2019). However, NRbCMs do not have a clear T-tubule structure and are not well suited for standard electrophysiological patch clamp techniques to evaluate the functions of sarcolemma ion channels. Therefore, we developed and characterized a novel rabbit cardiomyocyte model, namely, cultured 3-week-old ventricular cardiomyocytes (3wRbCMs), which have differentiated T-tubules, fully functional ion channels that generate an AP similar to adult rabbit myocytes, and thus can be used to investigate molecular mechanisms of cardiac arrhythmia at the cellular scale.

## Materials and Methods

### Animals

All animal experiments and procedures were approved by the Rhode Island Hospital Institutional Animal Care and Use Committee. In our breeding scheme of New Zealand White rabbits, we get 1 L every week for 2 weeks and then skip 1 week. The litter size ranges from 6 to 10 pups. Three-week-old ventricular cardiomyocytes (3wRbCMs) are isolated from the hearts of rabbits of 21–27 days of age (80% of them were 23–25 days old), both sexes, with standard enzymatic techniques using the Langendorff-based retrograde perfusion method. In each experiment, we used a minimum of four rabbits. The rabbits were administered with buprenorphine (0.03 mg/kg SQ), ketamine (100 mg/kg IM), xylazine (15 mg/kg IM), pentobarbital sodium (65 mg/kg IP), and heparin (1,000 U/kg IP). Briefly, the heart was removed from euthanized rabbits and perfused for 5–7 min (5 ml/min) with a nominally Ca^2+^-free solution containing (in mmol/L): 140 NaCl, 4.4 KCl, 1.5 MgCl_2_, 0.33 NaH_2_PO_4_, 5 HEPES, 16 taurine, 5 pyruvic acid, and 7.5 glucose. Subsequently, the heart was perfused for around 7 min with the same solution to which 0.5 mg/ml collagenase type I (Worthington Biochemical), and 0.1% BSA (Sigma, Cat# A7638-25G) were added. The whole ventricle was cut-off and minced, and the cells were dispersed with a pipette in a solution containing (in mmol/L): 45 KCl, 65 K-glutamate, 3 MgSO_4_, 15 KH_2_PO_4_, 16 taurine, 10 HEPES, 0.5 EGTA, 10 glucose (pH 7.3), and 10% BSA was added. The cell suspension was filtered through a 100-μm nylon mesh and sat down for half an hour. Cells were centrifuged, resuspended in Dulbecco’s Modified Eagle Medium (DMEM) (1 g glucose/l; 25 mM HEPES), supplemented with 5% heat-inactivated FBS and antibiotics, plated on laminin-coated (20 μg/ml) cover glasses or tissue culture dishes for further culturing. On average, we got 20% live cells. After 1–3 h, the medium was replaced to remove dead and non-attached cells.

### Cell Culture and Adenoviral Transduction

To culture 3wRbCMs for up to 48 h in DMEM, supplemented with 5% heat-inactivated FBS, we added 0.5 μM cytochalasin D to preserve the structure and function of cells during culture as previously described for adult rat cardiomyocytes (Tian et al., 2012). In the past, we have used and optimized liposome-mediated transfection, viz. lipofectamine 2000, on 3-day-old neonatal rabbit cardiomyocytes and achieved transfection rates of approximately 15% (Organ-Darling et al., 2013). However, 3wRbCMs like adult cardiomyocytes were completely resistant to liposome-mediated transfection (data not shown). Instead, we transduced 3wRbCMs with different amounts of adenovirus expressing cardiac cytomegalovirus enhanced myosin light chain (CMV-MLC) promoter-driven humanized *Renilla* Green Fluorescent Protein (GFP) with a multiplicity of infection (MOI) from 1 to 100 (Moshal et al., 2019). To minimize any unspecific effects caused by high MOIs, we decided to use an MOI of 10, which resulted in an approximately 90% infection rate after 48 h in culture.

### Cellular Morphology

For whole-cell imaging, we used a Nikon A1R confocal microscope, and all images were taken as Z-stacks. Wheat germ agglutinin staining (Alexa Fluor 594 conjugate) showed T-tubule structures, while anti-alpha-actinin antibody (Abcam, ab9465) detected Z-lines as the lateral boundaries of sarcomeres.

For high resolution cell imaging, we used a Philips 410 transmission electron microscope equipped with a 1k × 1k Advantage HR CCD camera. Freshly isolated 3wRbCMs were fixed with 0.15 M cacodylate buffer containing 2.5% glutaraldehyde, 2% paraformaldehyde, and 2 mM CaCl_2_. Cells were post-fixed, processed, and ultra-thin sections (80 nm) were prepared as described elsewhere (Russell et al., 2017).

### Immunoblot Analysis

Immunoblots were carried out as follows: freshly isolated or cultured cardiomyocytes were washed in PBS, centrifuged, and lysed in a suitable volume of RIPA buffer (Boston BioProducts) containing a Protease/Phosphatase Inhibitor Cocktail (Cell Signaling Technology). After sonication, extracts were rotated for 90 min at 4°C, centrifuged, and supernatants transferred to fresh tubes. After protein quantification, 40–80 μg of protein, heat-denatured (15 min at 60°C) in 4x Laemmli Sample Buffer (Bio-Rad; final concentration: 2x Sample Buffer; 100 mM DTT), were loaded per lane for SDS-PAGE. Protein was transferred to PVDF membranes, which were afterward blocked in 3% BSA. Membranes were incubated with the following antibodies overnight: rabbit anti-Cav1.2 (Alomone; ACC-003; 1:1,000); mouse anti-glyceraldehyde 3-phosphate dehydrogenase (GAPDH) (Thermo Fisher; 39-8600; 1:3,000); rabbit anti-human Ether-à-go-go-Related Gene (HERG) (Alomone; APC-062; 1:1,000); rabbit anti-Kir2.1 (Alomone; APC-026; 1:1,000); rabbit anti-Kv4.3 (Alomone; APC-017; 1:1,000); rabbit anti-Nav1.5 (Alomone; ASC-005; 1:1,000); rabbit anti-KCNQ1, polyclonal (Alomone; APC-022 1:1000); goat anti-Kv4.3, polyclonal (Santa Cruz Biotechnology; sc-11686; 1:300); and mouse anti humanized *Renilla* GFP, monoclonal (Agilent; 240141; 1:2,000). Suitable secondary HRP-conjugated antibodies (Thermo Fisher) were used at 1:10,000. Signals were detected using with SuperSignal West Pico PLUS Chemiluminescent Substrate (Thermo Fisher) and the ChemiDoc MP Imaging System (Bio-Rad). Protein band quantification was done with Image Lab software (Bio-Rad). Respective total channel protein expressions were normalized to GAPDH levels (see Supplementary Figures 1–9). For illustrative purposes (same protein samples), some of the images for GAPDH and GFP are reused in various (supplemental) figure panels.

### Electrophysiological Recordings and Data Analysis

**FIGURE 1:**
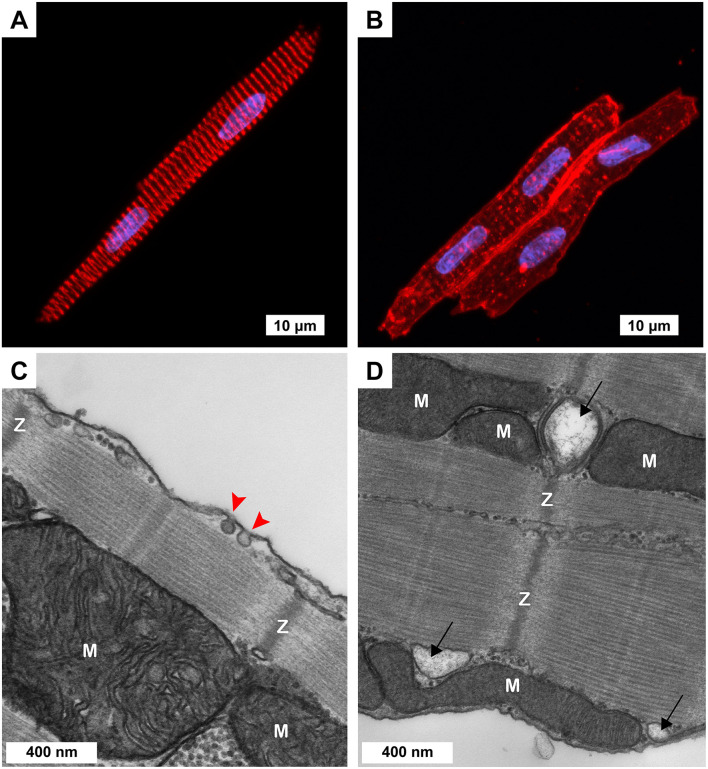
Cellular morphology of freshly isolated 3-week-old ventricular myocytes (3wRbCMs). **(A,B)** Representative confocal Z-stack images of 3wRbCMs. **(A)** Binuclear myocyte stained for α-actinin. Red, α-actinin; blue, DAPI. Scale bar, 10 μm. **(B)** Binuclear myocytes show cell membrane and T-tubules stained with wheat germ agglutinin (WGA). Red, WGA; blue, DAPI. Scale bar, 10 μm. **(C,D)** Representative transmission electron micrographs of 3wRbCMs. Surface-sarcolemmal caveolae are indicated by red arrows; T-tubules – by black arrows; Z-lines – by “Z”; mitochondria – by “M.” Scale bars, 400 nm.

In electrophysiological experiments, we used both freshly isolated and cultured single 3wRbCMs (Figure 1A). Some cells were transduced with adenovirus with an MOI of 10 to express GFP and were cultured between 44 and 50 h prior to performing electrophysiological recordings. GFP expression in these cells was verified by its fluorescence before each recording. All experiments were conducted in the whole-cell configuration at 35–37°C with Axopatch-200B, Digidata 1440A, and pClamp 10 software (Molecular Devices). After the cell membrane was broken by suction, the cell membrane capacitance and series resistance were compensated by 70–80% in the voltage clamp-mode. Tyrode solution of (in mM) 140 NaCl, 5.4 KCl, 1.8 CaCl_2_, 1 MgCl_2_, 0.33 NaH_2_PO_4_, 7.5 glucose, and 5 HEPES, with pH 7.4 adjusted with NaOH, was used as a standard bath solution. The pipette solution was modified to accommodate the measurement of a particular current of interest as described below, while the pipette resistance was in the range from 1.5 to 4 MΩ. The currents in each cell were normalized to the appropriate cell capacitance.

To record AP, the bath was perfused with Tyrode solution (as described above). The pipette solution contained (in mM): 90 potassium aspartate, 50 KCl, 5 NaCl, 1 MgCl_2_, 10 HEPES, 5 MgATP, and 0.1 GTP Tris, with pH 7.2 which was adjusted with KOH. AP was activated in the current clamp mode by a 3 ms injection of a depolarizing current, which was 20% higher than the threshold of AP activation. The AP stimulation rate was 0.25 Hz, while the voltage output was filtered by a low-pass filter with a cut-off frequency of 10 kHz and sampled at 20 kHz.

For sodium current (*I*_Na_) measurements, the bath solution contained (in mM): 5 NaCl, 140 TEA-Cl, 1 CaCl_2_, 1 MgCl_2_, 0.2 CdCl_2_, 10 HEPES, and 5 D-glucose, with pH adjusted to 7.4 with CsOH. While the pipette solution contained (in mM): 5 MgATP, 80 CsCl, 80 Cs aspartate, 1 MgCl_2_, 1 CaCl_2_, 11 EGTA, and 10 HEPES, with pH which was adjusted to 7.3 with CsOH. The holding potential was −120 mV, and 220 ms test pulses were from −100 to +40 mV in 10 mV increments. The current was filtered by a low-pass filter with a cut-off frequency of 10 kHz and sampled at 20 kHz.

The long-lasting calcium current (*I*_Ca,L_) was recorded in Tyrode solution, and the pipette solution contained (in mM): 105 CsCl, 10 TEA-Cl, 4 MgCl_2_, 10 EGTA, 10 HEPES, 4 Mg-ATP, and 10 Na_2_-phosphcreatine, with pH 7.2 which was adjusted with CsOH. The *I*_Ca,L_ current was activated by 300 ms depolarizing pulses from −50 mV holding potential to +60 mV, with 10 mV increments. The current was filtered by a low-pass filter with a cut-off frequency of 2 kHz and sampled at 10 kHz.

*I*_to_ was measured in modified Tyrode solution with zero sodium (in mM): 140 *N*-methyl-D-glucamine, 5.4 KCl, 1 CaCl_2_, 1 MgCl_2_, 5 HEPES, 7.5 D-glucose, and 0.2 CdCl_2_ which was added to suppress *I*_Ca,L_, with pH which was adjusted to 7.4 with HCl. The pipette solution contained (in mM): 110 K-aspartate, 20 KCl, 1 CaCl_2_, 1 MgCl_2_, 10 HEPES, 10 BAPTA, and 4 K_2_ATP, with pH which was adjusted to 7.2 with KOH. The holding potential was −80 mV, and the testing depolarizing steps were from −40 to +60 mV in 10 mV increments. The amplitudes for *I*_Na_, *I*_Ca,L_, and *I*_to_ were determined as their current peak values relative to the corresponding tail values at the end of a test pulse.

*I*_K1_ measurements were performed in slightly modified (Liu et al., 2020) Tyrode solution which contained (in mM): 135 NaCl, 5.4 KCl, 1.8 CaCl_2_, 1 MgCl_2_, 0.33 NaH_2_PO_4_, 10 glucose, and 10 HEPES, with a pH of 7.4 which was adjusted with NaOH. Nifedipine (1 μM) was added to block *I*_Ca,L_. The pipette resistance was 1.5–3 MΩ when filled with a solution containing (in mM): 140 KCl, 1 MgCl_2_, 10 EGTA, 5.0 K_2_ATP, and 5 HEPES, with pH of 7.3 which was adjusted with KOH. Holding potential was −40 mV and 400 ms voltage steps from −120 to +10 mV in 10 mV increments were applied at a 0.5 Hz rate. Capacitance and 70–80% of series resistance were routinely compensated. The sampling frequency was 10 kHz, and the −3 dB cut-off frequency was 5 kHz. The *I*_K1_ amplitude was determined as the end of the testing voltage pulse. The currents were normalized to cell membrane capacitance.

Rapid and slow components of the delayed rectifier potassium current (*I*_Kr_ and *I*_Ks_) recordings were performed in modified Tyrode solution (in mM): 140 NaCl, 1.5 KCl, 0.33 Na_2_HPO_4_, 0.2 CaCl_2_, 0.2 MgCl_2_, 0.2 CdCl_2_, 5 HEPES, and 7.5 D-glucose, with pH which was adjusted to 7.4 with NaOH. The pipette resistance was 2–4 MΩ when filled with intracellular solution (in mM): 130 KCl, 0.36 CaCl_2_, 5 EGTA, 5 HEPES, 5 D-glucose, 5 Mg-ATP, 5 Na_2_-phosphocreatine, and 0.25 Na_2_-GTP, with pH of 7.2 which was adjusted with KOH. *I*_Kr_ recording started at −40 mV holding potential followed by a series of 3 s test pulses that were applied in 10 mV increments to a maximum membrane potential of +40 mV. Then, the experiments were repeated in the presence of the specific *I*_Kr_ blocker, E-4031 (5 μM, Abcam). The difference, E-4031-sensitive current, was considered as *I*_Kr_. The *I*_Kr_ amplitude was measured as the peak of the E-4031-sensitive tail current after the end of each depolarizing test pulse. Similarly, *I*_Ks_ was identified as chromanol-293b- (30 μM) sensitive current in the presence of 5 μM E-4031. *I*_Ks_ was measured at the end of each depolarizing pulse. Capacitance and 70–80% of series resistance were routinely compensated. The sampling frequency was 20 kHz, and the −3 dB cut-off frequency was 5 kHz. The currents were normalized to appropriate cell membrane capacitance.

### Confocal Microscopy Measurements of Ca^2+^ Transients

Intracellular Ca^2+^ cycling in freshly isolated 3wRbCMs was monitored by a Leica SP2 confocal laser scanning system equipped with a ×63 1.4 NA oil-immersion objective in linescan mode using the Ca^2+^-sensitive indicator Fluo-4 (Thermo Fisher). Cells were loaded with Fluo-4 for 20 min, and after 20 min de-esterification, the dye was excited with the 488 nm line of an argon laser. Emission was collected at 500–541 nm. Cardiomyocytes were studied in Tyrode solution (in mM, NaCl 140, KCl 5.4, CaCl_2_ 1.8, MgCl_2_ 1, Glucose 5.6, HEPES 10, and pH 7.3) and with 100 nM isoproterenol, a β-adrenergic receptor agonist. Cells were paced by electric field stimulation at 0.5 Hz using extracellular platinum electrodes. To assess the sarcoplasmic reticulum (SR) Ca^2+^ load and Ca^2+^ clearance rates, 350 μl of 10 mM caffeine were applied to 150 μl bath solution at the end of each experiment, which resulted in a 7 mM final caffeine concentration. The sodium–calcium exchanger (NCX) activity was estimated by measuring the decay rate of caffeine-induced Ca^2+^ transients (*k*_*caff*_). Sarcoplasmic/endoplasmic reticulum (ER) Ca^2+^-ATPase (SERCA) activity (*k*_*SR*_) was estimated as the second component of two-exponential decay fitting of electric field induced Ca^2+^ transients when the first exponential component time constant was set as 1/*k*_*caff*_, as it was described previously (Cooper et al., 2013).

### Statistical Analysis

The quantifications of western blot data are presented as mean ± SD, while all other data are presented as mean ± SEM to improve visibility of the I–V curves. Statistical analyses were performed with GraphPad Prism 8.0 (GraphPad Software Inc.). The statistical significance was evaluated using an unpaired Student’s *t*-test. A value of *p* < 0.05 was considered statistically significant. The Boltzmann fitting curves were generated with OriginPro 2019 (OriginLab Corporation).

## Results

### Morphological Characterization of 3-Week-Old Ventricular Cardiomyocytes

Acutely isolated 3wRbCMs revealed completely organized sarcomeres with α-actinin staining (Figure 1A). To stain the surface of cell membranes and T-tubules, we used wheat germ agglutinin (WGA). WGA staining showed a well-structured T-tubule system in these cells (Figure 1B). However, the left cell in Figure 1B has a more developed T-tubule system than the right cell. We have also performed similar WGA and α-actinin staining of cardiomyocytes prepared from younger and older rabbit hearts (data not shown) and found that T-tubules can first be seen as early as 11-day-old rabbit cardiomyocytes. We concluded that cardiomyocytes prepared from rabbits as early as 3 weeks of age resembled adult rabbit cardiomyocytes with respect to T-tubule structure, striation, and rod shape. Of note, in some of our electrophysiological experiments of cultured (48 h) 3wRbCMs, we noticed contraction of these cells during stimulation and during leak of calcium into the cell due to patch seal leak, which confirms the ability of these cells to contract and thus having functional sarcomeres.

Morphological maturity of 3wRbCMs was confirmed by transmission electron micrographs of 3wRbCMs (Figures 1C,D), which display distinctly visible surface-sarcolemmal caveolae, T-tubules, Z-lines, and mitochondria. Therefore, we decided to characterize basic cardiac ion currents and corresponding ion channel proteins in acutely isolated and cultured 3wRbCMs. This would allow establishing a new assay to estimate the effects of various molecules of interest expressed through viral infection on cellular electrophysiology.

### Action Potential and Ca^2+^ Transients

**FIGURE 2:**
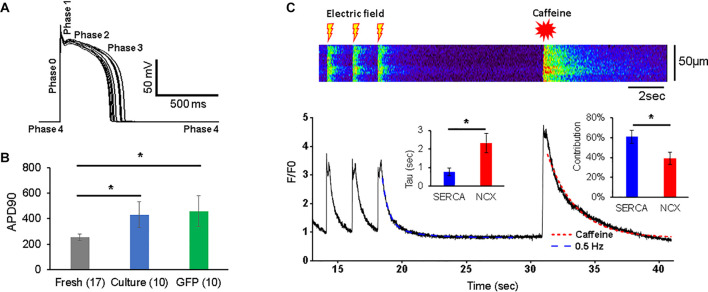
Action potentials (APs) and Ca^2+^ transients in 3wRbCMs. **(A)** Superposition of APs of a representative freshly isolated 3wRbCM that were evoked by 3 ms depolarizing current pulses at 0.25 Hz. Five phases of the AP are indicated. **(B)** Action potential duration at 90% repolarization level (APD90) in freshly isolated (fresh), 48 h cultured, non-transduced (culture), and 48 h cultured, GFP-transduced (GFP) cells. **(C)** Representative Ca^2+^ transients in a freshly isolated 3wRbCM in the presence of 100 nM isoproterenol (ISO). Initially, the transients were evoked by electric field stimulation at 0.5 Hz, while the very last transient was evoked by application of 7 mM final caffeine concentration. Only the last three transients prior to the addition of caffeine are shown. Left insert shows averaged values of exponential decay time constants (Tau) for Ca^2+^ transients caused by sodium–calcium exchanger (NCX) (in the presence of caffeine) and sarcoplasmic/endoplasmic reticulum Ca^2+^-ATPase (SERCA). The right insert shows relative contributions of SERCA and NCX to the total Ca^2+^ clearance after the electric stimulation (*N* = 7, *n* = 10). The symbol * corresponds to *p* < 0.05.

The membrane potential and Ca^2+^-transients were measured as described in the methods section. The resting potential in freshly isolated 3wRbCMs was −78.8 ± 0.4 mV (*n* = 17), −74 ± 2 mV (*n* = 10) in 48 h cultured non-transduced cells, and −78.7 ± 0.8 mV (*n* = 10) in 48 h cultured GFP-transduced cells. Thus, the resting potential in cultured, non-transduced cells was slightly but significantly depolarized compared to both freshly isolated and cultured GFP-transduced cells (*p* < 0.05). Cells were stimulated at 0.25 Hz, and the average AP duration (APD90) was measured at the 90% repolarization level relative to the peak. The average APD90 in cultured, non-transduced cells (430 ± 103 ms) and in 48 h cultured GFP-transduced cells (457 ± 121 ms) was significantly longer (*p* < 0.05) than the APD in freshly isolated 3wRbCMs (253 ± 24 ms) (Figure 2B). However, APD50 values for these three cell groups were not significantly different (Supplementary Figure 10). This implies electrical remodeling of 3wRbCMs during culturing for 48 h and requires further studies of the ionic currents and corresponding channel proteins. In addition, we found that the cell membrane capacitance during 48 h of culturing dropped significantly (*p* < 0.01) from 49.1 ± 3.4 pF (in fresh cells) to 30.6 ± 2.2 pF (in cultured, non-transduced cells) and to 29.9 ± 2.6 pF (in cultured GFP-transduced cells), i.e., by 38 and 39%, respectively, which is similar to 42% capacitance reduction found in cultured adult rabbit cardiac myocytes (Mitcheson et al., 1996).

The Ca^2+^ transients were evoked with a 0.5 Hz electric field stimulation in freshly isolated 3wRbCMs (Figure 2C). In order to determine relative contributions of the NCX and SR/ER Ca^2+^-ATPase (SERCA) to Ca^2+^ clearance in 3wRbCMs, 7 mM of final caffeine concentration was applied after the last electric field stimulation. We did not observe any significant changes in Ca^2+^ clearance rate in acutely isolated 3wRbCMs as compared to adult cardiomyocytes (Terentyev et al., 2014) suggesting intact calcium cycling in the former. Although intracellular calcium transients might be affected by mitochondrial and lysosomal calcium uptakes (Trollinger et al., 2000; Raffaello et al., 2016; Yeh et al., 2020; Negri et al., 2021), for simplicity, we assumed that the caffeine-evoked transient decay is determined only by NCX extrusion of Ca^2+^ and it could be fitted with a one-exponential decay function. The adjusted *R*^2^ value for one exponential fit was 98% (Figure 2C). Thus, based on a one-exponential fit of the caffeine-only evoked transients, we determined the time constant corresponding to the NCX calcium clearance.

In order to determine the SERCA-dependent component of Ca^2+^ clearance, we fit the electrically evoked Ca^2+^ transients with a two-exponential decay function. In one exponent, we fixed the time constant equal to the value of NCX-dependent decay found from the caffeine-evoked transient, while in another exponent, the time constant (attributed to SERCA) was determined by the two-exponential fit (Figure 2C). These time constants gave us Ca^2+^ clearance rates for SERCA and NCX as 2.3 ± 0.5 s^–1^ and 0.75 ± 0.20 s^–1^, respectively (*n* = 10). In addition, the amplitudes of the fits allowed us to determine the relative contributions of SERCA and NCX to Ca^2+^-clearance after electric field stimulation, which are 61 ± 6 and 39 ± 6%, respectively.

### Depolarizing Currents and Corresponding Channels

**FIGURE 3:**
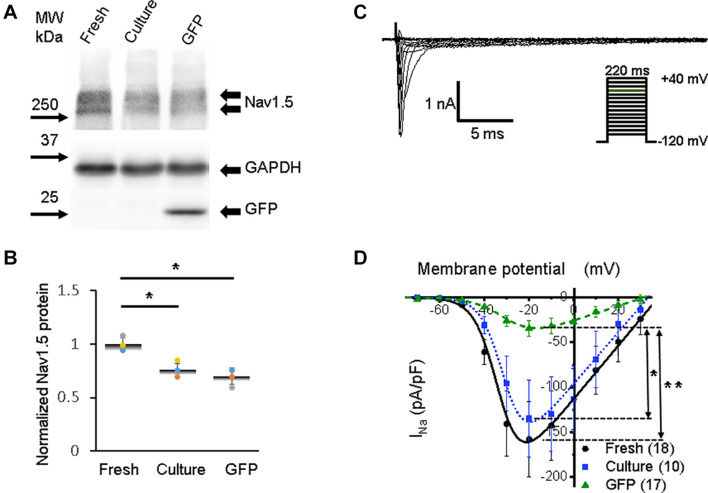
Sodium channel protein (Nav1.5) and sodium current (*I*_Na_) in 3wRbCMs. **(A)** Western blot of Nav1.5, tetrodotoxin-resistant voltage-gated sodium channel subunit alpha 5 in freshly isolated (fresh), 48 h cultured, non-transduced (culture), and 48 h cultured, Green Fluorescent Protein (GFP)-transduced cells. For illustrative purposes (same protein samples), the images for GAPDH and GFP are reused in Figure 7A (Kv11.1). **(B)** Quantification of the western blots (*N* = 4) shows significant downregulation of total Nav1.5 protein during 48 h of culturing with and without GFP transduction compared to freshly isolated cells. **(C)** Representative *I*_Na_ traces of a freshly isolated 3wRbCM. The voltage clamp protocol is shown in the insert. **(D)** Cumulative I–V curves of freshly isolated cells (black circles, *N* = 4, *n* = 18), 48 h cultured, non-transduced cells (blue squares, *N* = 4, *n* = 10), 48 h cultured GFP-transduced cells (green triangles, *N* = 4, *n* = 17). The symbols * and ** correspond to *p* < 0.05 and *p* < 0.01, respectively.

The activation of voltage-dependent sodium channels produces a rapid AP upstroke (phase 0), while activation of the L-type voltage-dependent Ca^2+^ current (*I*_Ca,L_) in larger animals is required to maintain the plateau phase of AP (phase 2) (Bartos et al., 2015). The shape of the AP upstroke (Figure 2A) suggests a robust inward *I*_Na_. Our western blot analysis demonstrated significant expression of Nav1.5 in freshly isolated, 48 h cultured non-transduced, and 48 h cultured GFP-transduced 3wRbCMs (Figure 3A). Quantification of the western blots (*N* = 4) shows downregulation of total Nav1.5 protein during 48 h of culturing with and without GFP transduction compared to freshly isolated cells (*p* < 0.05). We have recorded the whole-cell *I*_Na_ at a reduced extracellular sodium concentration in three groups: freshly isolated, 48 h cultured, non-transduced, and 48 h cultured GFP-transduced 3wRbCMs (Figures 3C,D), with *I*_Na_ peak values at −20 mV equal to −158 ± 42 pA/pF (*n* = 18), −135 ± 43 pA/pF (*n* = 10), and −32 ± 9 pA/pF (*n* = 17), respectively. The *I*_Na_ peak in GFP-transduced cells was significantly smaller than the peaks in both freshly isolated cells (*p* < 0.01), and 48 h cultured, non-transduced cells (*p* < 0.05). This observation implies that GFP expression alone may cause a significant reduction of *I*_Na_ during 48 h of culturing 3wRbCMs. However, statistically significant reduction of the expression of total Nav1.5 protein in both cultured cell groups compared to the freshly isolated group indicates that reduction of *I*_Na_ in GFP-transduced cells is likely caused by additional nuclear and cellular GFP effects (Agbulut et al., 2007; Wallace et al., 2013).

**FIGURE 4:**
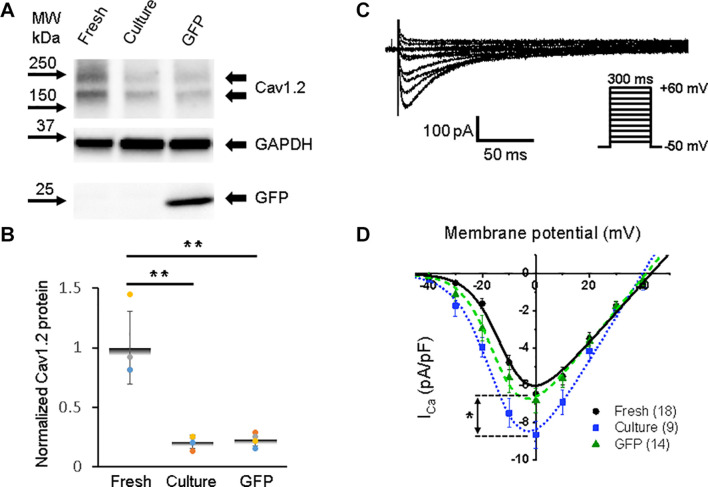
Calcium channel protein Ca_*V*_1.2 and L-type calcium current (*I*_Ca,L_) in 3wRbCMs. **(A)** Western blot of voltage-gated calcium channel subunit alpha 1C (Ca_*V*_1.2) in freshly isolated, 48 h cultured, non-transduced, and 48 h cultured, GFP-transduced cells. **(B)** Quantification of the western blots (*N* = 4) shows significant downregulation of total Ca_*V*_1.2 protein during 48 h of culturing with and without GFP transduction compared to freshly isolated cells. **(C)** Representative *I*_Ca,L_ traces of a freshly isolated 3wRbCM. The voltage clamp protocol is shown in the insert. **(D)** Cumulative I–V curves of freshly isolated cells (black circles, *N* = 4, *n* = 18), 48 h cultured, non-transduced cells (blue squares, *N* = 4, *n* = 9), 48 h cultured, GFP-transduced cells (green triangles, *N* = 4, *n* = 14). The symbols * and ** correspond to *p* < 0.05 and *p* < 0.01, respectively.

A distinct expression of the alpha 1C subunit of L-type voltage-dependent calcium channel (also known as Cav1.2) was found in freshly isolated, 48 h cultured, non-transduced, and 48 h cultured GFP-transduced 3wRbCMs (Figure 4A), yet with a significant reduction (*p* < 0.01) of the total protein expression in cultured cells with and without GFP transduction compared to the freshly isolated cells (Figure 4B). However, there was no reduction in L-type calcium currents in cultured cells with and without GFP expression compared to freshly isolated cells (Figures 4C–D). Moreover, there is a 34% increase in maximal *I*_Ca,L_ (*p* < 0.05, Supplementary Table 1) in the cultured, non-transduced cells compared to the freshly isolated cells, which is similar to small changes of *I*_*Ca*_ in cultured mouse sinoatrial node myocytes (St Clair et al., 2015). This increase in normalized *I*_*Ca*_ is associated with the reduction of cell membrane capacitance by 38% in cultured cells and supports the protective effect of cytochalasin D on the T-tubule system (Chung et al., 2008).

Both sodium and calcium current-voltage relationships were fitted with OriginPro 2019 Boltzmann I–V function (Figures 3D, 4D):


$$\text{I}=\left(\varphi -E_{\mathrm{rev}}\right)\,g_{\max}/\left(1+\exp\,\left(-\,\left(\varphi -V_{1/2}\right)/V_{z}\right)\right)$$

The best fit of our *I*_Na_ data in freshly isolated 3wRbCMs (Figure 3D) gave us *g*_*Na,max*_ = 3.0 ± 0.3 nS/pF, *V_1/2,Na_* = −33 ± 2 mV, and *V*_*z,Na*_ = 5.2 ± 0.5 mV. In 48 h cultured, non-transduced cells: *g*_*Na,max*_ = 2.9 ± 0.3 nS/pF, *V_1/2,Na_* = −31 ± 1 mV, and *V*_*z,Na*_ = 5.0 ± 0.3 mV. In 48 h cultured GFP-expressing cells: *g*_*Na,max*_ = 0.8 ± 0.1 nS/pF, *V_1/2,Na_* = −29 ± 2 mV, and *V*_*z,Na*_ = 7.0 ± 0.6 mV. The major difference in the fitting parameters between these three groups was in *g*_*Na,max*_, i.e., its reduction in GFP-expressing cells. The latter is probably caused by the decrease in the number of functional sodium channels on the plasma membrane in GFP-transduced 3wRbCMs in culture.

We should mention that *g*_*Na,max*_ is relatively small even in freshly isolated 3wRbCMs, which is due to low extracellular sodium concentration that is 28 times smaller than the physiological sodium concentration. We have used this low sodium concentration specifically to reduce *I*_Na_ and thus minimize the voltage clamp error due to non-zero access resistance even with series resistance compensation.

The best fit parameters for *I*_Ca,L_ in freshly isolated cells (Figure 4D) gave us *g*_*Ca,max*_ = 161 ± 16 pS/pF, *V_1/2,Ca_* = −11 ± 2 mV, and *V*_*z,Ca*_ = 5.9 ± 0.6 mV. For 48 h cultured non-transduced cells the best fitting parameters were: *g*_*Ca,max*_ = 237 ± 20 pS/pF, *V_1/2,Ca_* = −14 ± 1 mV, and *V*_*z,Ca*_ = 6.7 ± 0.7 mV, while for 48 h cultured GFP-transduced cells: *g*_*Ca,max*_ = 178 ± 11 pS/pF, *V_1/2,Ca_* = −14 ± 1 mV, and *V*_*z,Ca*_ = 5.9 ± 0.4 mV. There are no significant differences between the parameters in these groups of cells. However, we should mention that *I*_Ca,L_ variability is larger in the cultured cells compared to the freshly isolated cells.

### Repolarizing Currents and Corresponding Channels

**FIGURE 5:**
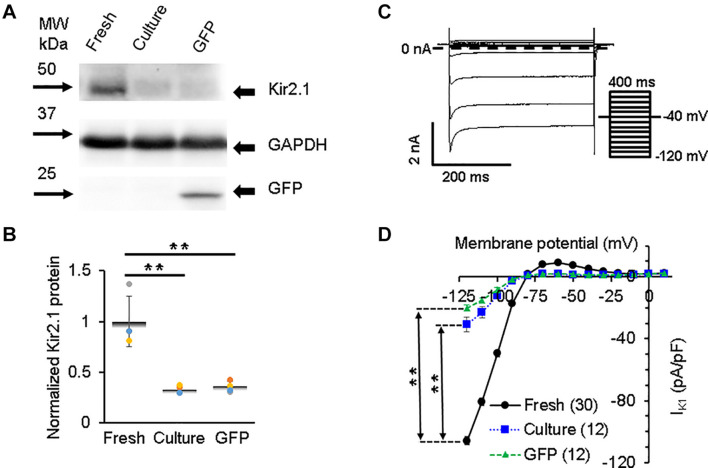
Inwardly rectifying potassium current (*I*_*K1*_) and corresponding Kir2.1 protein in 3wRbCMs. **(A)** Western blot of Kir2.1, inwardly rectifying channel subunit in freshly isolated, 48 h cultured, non-transduced, and 48 h cultured, GFP transduced cells. **(B)** Quantification of the western blots (*N* = 4) shows significant downregulation of total Kir2.1 protein during 48 h of culturing with and without GFP transduction compared to freshly isolated cells. **(C)** Representative *I*_*K1*_ traces in a freshly isolated 3wRbCM. Voltage clamp protocol is shown in the insert. **(D)** Cumulative *I*_*K1*_ I–V curves of freshly isolated, 48 h cultured, non-transduced, and 48 h cultured, GFP-transduced cells. The symbol ** corresponds to *p* < 0.01.

Before the AP upstroke and after AP repolarization (phase 4 in Figure 2A), the resting potential is maintained close to the potassium Nernst equilibrium potential mostly by inward rectifier current (*I*_K1_), which opens at negative membrane potentials (Liu et al., 2004). *I*_K1_ also contributes to the terminal phase of repolarization of AP (phase 3) (Nerbonne and Kass, 2005; Cordeiro et al., 2015). We found a strong expression of total inward rectifier potassium channel protein (Kir2.1) in freshly isolated 3wRbCMs (Figure 5A) but significantly reduced (*p* < 0.05) Kir2.1 expression in cultured cells with and without GFP transduction (Figures 5A,B). These data correlate with comparable changes in *I*_K1_, with reduced *I*_K1_ (*p* < 0.01) in both cultured cell groups (Figures 5C,D) and can explain part of the aforementioned APD90 prolongation (Figure 2B).

**FIGURE 6:**
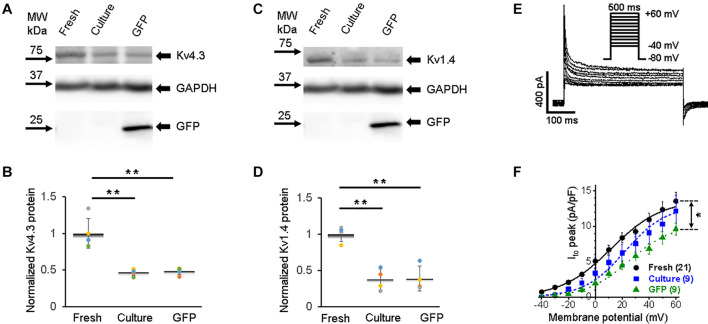
Transient outward potassium current (*I*_to_) and corresponding Kv4.3 and Kv1.4 proteins in 3wRbCMs. **(A)** Western blot of Kv4.3, a prominent alpha subunit that encodes the fast component of I_to_ in freshly isolated, 48 h cultured non-transduced, and 48 h cultured GFP transduced cells. **(B)** Quantification of the western blots (*N* = 5) shows significant downregulation of the total of Kv4.3 protein during 48 h culturing with and without GFP transduction compared to the freshly isolated cells. For illustrative purposes (same protein samples), the images for glyceraldehyde 3-phosphate dehydrogenase (GAPDH) and GFP are reused in Figure 6B (Kv1.4) and 8A (Kv7.1). **(C)** Western blot of Kv1.4 that encodes the slow component of I_to_ in freshly isolated, cultured, and GFP-transduced cultured cells. For illustrative purposes (same protein samples), the images for GAPDH and GFP are reused in Figure 6A (Kv4.3) and 8A (Kv7.1). **(D)** Quantification of the western blots (*N* = 4) shows significant downregulation of the total of Kv1.4 protein during 48 h culturing with and without GFP transduction compared to the freshly isolated cells. **(E)** Representative *I*_to_ traces in freshly isolated 3wRbCMs show two-exponential decay with 7 and 117 ms time constants (see section “Results”). **(F)** Cumulative I–V curves of freshly isolated, 48 h cultured non-transduced, and 48 h cultured GFP transduced cells. The symbols * and ** correspond to *p* < 0.05 and *p* < 0.01, respectively.

The rapid early repolarization (phase 1 in Figure 2A) immediately follows the AP upstroke, and it is produced by the transient outward K^+^ current (*I*_to_). While the delayed repolarization of AP (phase 3) is mostly shaped by delayed-rectifier potassium currents with slow and rapid activation kinetics, namely, *I*_Ks_ and *I*_Kr_, respectively (Bartos et al., 2015). The pore-forming subunits of channels that underlie *I*_to_ in rabbits are derived from *KCND3* (Kv4.3), *KCND2* (Kv4.2), and *KCNA4* (Kv1.4) genes (Wang et al., 1999; Zicha et al., 2003; Nerbonne and Kass, 2005). *I*_to_ is also affected by Kv channel interacting protein (KChIP2) (Niwa and Nerbonne, 2010). In all three groups of 3wRbCMs, we found strong expression of the Kv4.3 subunit (Figure 6A), which has the largest contribution to the total *I*_to_ current in rabbit myocytes when inhibited by antisense oligo as compared to Kv4.2 or Kv1.4 subunits (Wang et al., 1999). Similar to all channels described above, cultured cells with and without GFP expression had reduced (*p* < 0.01) total protein levels of Kv4.3 compared to freshly isolated cells (Figure 6B). Likewise, the Kv1.4 subunit, which underlies a significant portion of *I*_to_ current in rabbits (Wang et al., 1999), is expressed in all three groups of 3wRbCMs (Figure 6C), and it is also significantly downregulated in both groups of the cultured cells (Figure 6D).

Similar to *I*_Na_ (Figure 3D), only GFP-expressing cells had a significantly lower *I*_to_ current amplitude (Figures 6E–F). The *I*_to_ current exhibited two-exponential inactivation and in freshly isolated 3wRbCMs at +40 mV, the fast-decaying component (τ_fast_ = 7 ± 1 ms) contributed 76 ± 3% to the total *I*_to_ amplitude, while the slow component (τ_slow_ = 117 ± 28 ms) contributed 24 ± 3% (*N* = 4, *n* = 17). It should be noted that both *I*_to,f_. and *I*_to,s_ contribute to these fast and slow inactivation components (Brahmajothi et al., 1999; Patel and Campbell, 2005). The studies of Kv1.4-encoded current (*I*_to.s_) are beyond the scope of this study.

**FIGURE 7:**
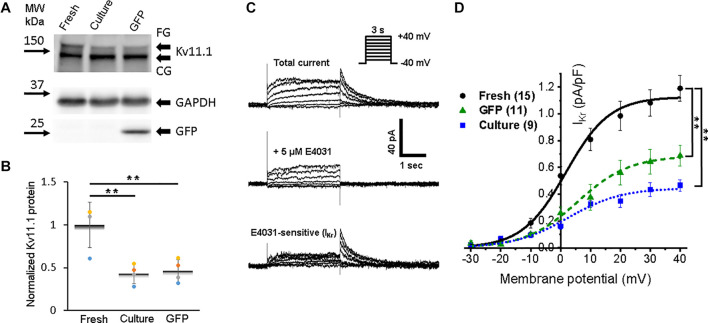
Expression of rabbit Ether-à-go-go-Related Gene (RERG) protein and a corresponding rapid component of the delayed rectifier potassium current (*I*_Kr_) in 3wRbCMs. **(A)** Western blot of fully glycosylated (FG) and core-glycosylated (CG) RERG (*Kcnh2*) in freshly isolated, 48 h cultured, non-transduced, and 48 h cultured, GFP-transduced 3wRbCMs. For illustrative purposes (same protein samples), the images for GAPDH and GFP are reused in Figure 3A (Nav1.5). **(B)** Quantification of the western blots (*N* = 4) reveals significant downregulation of total FG RERG protein during 48 h culturing with and without GFP transduction compared to freshly isolated cells. **(C)** Representative current traces before and after application of *I*_Kr_ blocker, E-4031, in a cultured, GFP-transduced 3wRbCM. Bottom traces represent their difference, i.e., *I*_Kr_. Voltage clamp protocol is shown in the insert. **(D)** Cumulative I–V curves of freshly isolated cells (black circles, *N* = 6, *n* = 15), 48 h cultured, non-transduced cells (blue squares, *N* = 4, *n* = 9), 48 h cultured, GFP-transduced cells (green triangles, *N* = 7, *n* = 11). The symbol ** corresponds to *p* < 0.01.

Accumulation of *I*_Kr_ and *I*_Ks_ currents persist during the plateau phase and play a crucial role in the AP repolarization phase 3. We found fully glycosylated (FG) and core-glycosylated (CG) rabbit Ether-à-go-go-Related Gene (RERG, Kv11.1) channel protein in all three groups of the cells (Figure 7A). However, the reduction (*p* < 0.01) of Kv11.1 expression in both cultured groups of 3wRbCMs was observed only for the FG protein (Figure 7B).

To identify *I*_Kr_, we used its specific blocker E-4031, and the tail peak of the E-4031 sensitive component was used as the measure of *I*_Kr_ (Figure 7C). The average *I*_Kr_ value at the +30 mV depolarizing pulse was equal to 1.1 ± 0.1, 0.44 ± 0.05, and 64 ± 0.09 pA/pF in freshly isolated, 48 h cultured, non-transduced, and 48 h cultured, GFP-transduced 3wRbCMs, respectively (Figure 7D). Thus, cultured, non-transduced, and GFP-transduced cells had both significantly smaller *I*_Kr_ current compared to *I*_Kr_ in freshly isolated cells (*p* < 0.01), which correlates with the western blot analysis (Figure 7B).

**FIGURE 8:**
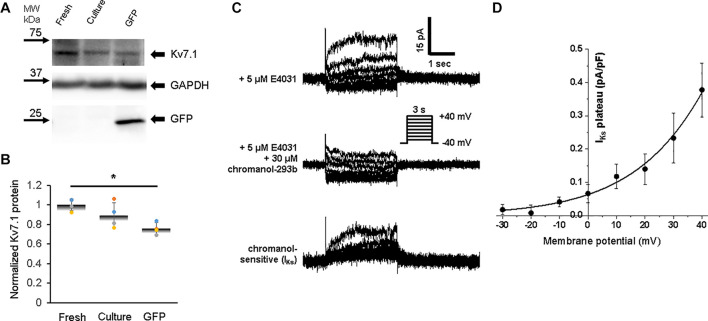
Expression of rabbit Kv7.1 protein and a corresponding slow component of the delayed rectifier potassium current (*I*_Ks_) in 3wRbCMs. **(A)** Western blot of Kv7.1 protein in freshly isolated, 48 h cultured non-transduced, and 48 h cultured GFP transduced cells. For illustrative purposes (same protein samples), the images for GAPDH and GFP are reused in Figure 6A (Kv4.3) and 6B (Kv1.4). **(B)** Quantification of the western blots (*N* = 4) shows significant downregulation of total Kv7.1 protein only in GFP-expressing cells after 48 h in culture (*p* < 0.05, *N* = 4). **(C)** Representative current traces before (top) and after application of *I*_Ks_ blocker, chromanol-293b (middle), in the presence of 5 μM E4031. Bottom traces correspond to their difference, i.e., *I*_Ks_. Voltage clamp protocol is shown in the insert. **(D)** Cumulative I–V curve of *I*_Ks_ plateau measured at the end of the depolarizing pulse (*N* = 4, *n* = 12). The symbol * corresponds to *p* < 0.05.

Expression of Kv7.1 (KvLQT1) protein, which corresponds to the slow component of the delayed rectifier potassium current, *I*_Ks_, was relatively weak in freshly isolated 3wRbCMs (Figure 8A). The 48 h culturing with and without GFP transduction leads to some decrease of Kv7.1 expression, but only GFP-expressing cells had a significant (*p* < 0.05) Kv7.1 decrease (Figure 8B).

Slow components of the delayed rectifier potassium current were identified in freshly isolated 3wRbCMs as the chromanol-293b-sensitive current in the presence of E-4031 (Figure 8C). We noticed that the chromanol-sensitive plateau current, *I*_Ks_ (Figure 8D), was about 3 times smaller than the *I*_Kr_ tail current (Figure 7D). *I*_Ks_ was undetectable in cultured cells with and without GFP expression even though the protein was still detectable. For example, in the presence of E-4031, the *I*_Ks_ tail current (after the depolarizing voltage step) was visible in a representative acutely isolated 3wRbCM (Figure 8C, top panel), but not in a GFP-transduced cell (Figure 7C, middle panel).

## Discussion

We were looking for a low-cost alternative to the adult rabbit cardiomyocyte model for our studies of molecular mechanisms of arrhythmia. Here, we describe the use of 3wRbCMs as an alternative model. Freshly isolated cells can be used to study the acute pharmacologic effects of drugs added to cells, whereas 3wRbCM cultures allow investigators to transduce cells to express molecules of interest or incubate cells with drugs to study their effect on various biochemical, biophysical, and electrophysiological characteristics of the myocytes. 3wRbCMs are also amenable to knockdown of protein expression using shRNA-expressing adenovirus (Moshal et al., 2019). WGA staining has revealed a fully developed T-tubule system in most cells, while α-actinin staining uncovered organized sarcomeres in 3wRbCMs (Figures 1A,B). Additionally, transmission electron microscopy revealed surface-sarcolemmal caveolae, T-tubules, Z-lines, and mitochondria (Figures 1C,D). The cells have exhibited APs with APD of 253 ± 24 ms (Figure 2A), which is relatively close to human ventricular APD90 of approximately 285 ms (Jost et al., 2005). Compared to isolated human ventricular cardiomyocytes (Jost et al., 2005), cultured 3wRbCMs display a similar upstroke shape and prominent notch (phase 1), but have a significantly shorter plateau (phase 2). 3wRbCMs exhibited Ca^2+^ transients (Figures 2B,C) with Ca^2+^ clearance rates of 2.3 s^–1^ for SERCA and 0.75 s^–1^ for NCX, which are similar to the corresponding rates of 3.2 s^–1^ and 0.47 s^–1^ found in adult rabbit cardiac myocytes (Moshal et al., 2019).

Freshly isolated and cultured 3wRbCMs have a complete set of ion channels required for a normal adult rabbit-like AP (Figures 3–8, Supplementary Figure 9, and Supplementary Table 1). However, during 48 h of culturing, expression of all studied channels decreases, which in most, but not all cases correlates with the changes in the corresponding ion currents. In our experimental conditions, the *I*_Na_ peak current in freshly isolated 3wRbCMs was equal to −158 ± 42 pA/pF, and 48 h cultured cells *I*_Na_ was similar (−135 ± 43 pA/pF) in non-transduced, but in GFP-transduced 48 h cultured cells, *I*_Na_ was significantly smaller (−32 ± 9 pA/pF) (Figure 3D). The latter indicates the decrease in functional sodium channels is caused by GFP transduction and consequent expression of GFP polypeptides, but not by the culturing itself. Since the cardiac sodium channel life cycle has multiple steps (biosynthesis, trafficking, anchoring, posttranslational modifications, recycling, degradation, etc.) (Dong et al., 2020) that could be affected during 48 h of culturing and/or by expression of GFP. Additional studies are required to identify the underlying cause for the changes in the expression and function of the sodium channel. Yet, 3wRbCMs exemplifies a remarkable system to investigate the regulation of *I*_Na_ by different factors (Turan et al., 2020).

Comparatively, in freshly isolated adult human atrial cells, the *I*_Na_ peak was found to be −47 pA/pF (Cai et al., 2011) or −84 pA/pF (Casini et al., 2019) while in human pediatric atrial cells, it was −32 pA/pF (Cai et al., 2011). The peak current values in canine adult ventricular myocytes ranged from −75 to −78 pA/pF, although in 2-week-old neonate canine ventricular myocytes, it was −45 pA/pF (Biet et al., 2012; Cordeiro et al., 2013). It should be noted that experimental conditions used to particularly measure the sodium currents vary significantly in different reports based on the preferences of the authors. Therefore, one cannot directly compare the data from different labs.

In contrast to *I*_Na_, the maximal amplitude of *I*_Ca,L_ was not changed in 48 h cultured, GFP-transduced 3wRbCMs compared to the fresh cells (Figure 4D). However, *I*_Ca,L_ in cultured, non-transduced cells slightly increased, which is not supported by the noticed decrease in total Cav1.2 expression (Figure 4B), but it is comparable to a small increase in *I*_*Ca*_ in cultured mouse sinoatrial node myocytes (St Clair et al., 2015). These apparent contradictions may arise due to the complexity of electrical remodeling. For example, the biphasic changes in the calcium current during the culturing of adult rabbit myocytes in “non-supplemented” media and monophasic changes in “supplemented” media (Figure 2d of Mitcheson et al., 1997). Nevertheless, the functional calcium channels are robust and stable during 48 h of culturing (Moshal et al., 2019), and they are not affected by overexpression of GFP. Our values of *I*_Ca,L_ (−7 to −9 pA/pF) in 3wRbCMs are comparable to previously reported maximal *I*_Ca,L_ in the range from −4 to −6 pA/pF found in adult (13 weeks old) rabbit cardiac ventricular myocytes (Kalik et al., 2017) and *I*_Ca,L_ of −7.5 pA/pF found in human ventricular myocytes (Jost et al., 2013). Of note, the persistence of *I*_*Ca*_ and small changes in *I*_to_ in cultured cells likely underlie the lack of prolongation of APD50 in cultured cells as compared to freshly dissociated myocytes (Supplementary Figure 10).

All studied potassium channel proteins (Kir2.1, Kv4.3, Kv1.4, Kv11.1, and Kv7.1) are clearly expressed in freshly isolated 3wRbCMs (Figures 5–8), while in cultured non-transduced and GFP-transduced cells, the expression of the channels is significantly decreased which underlie the significant prolongation of the APD90 (Supplementary Figure 10). Yet, all corresponding currents can be measured in cultured cells except for *I*_Ks_, which was very small even in the fresh cells. The reduction of Kir2.1 polypeptide expression and *I*_K1_ amplitude in cultured cells was significant and almost identical (Figure 5).

Although guinea pig left ventricular myocytes have a similar AP duration at 90% of the repolarization (APD90) (Beserra et al., 2020; Al-Owais et al., 2021) to the rabbit APD, they lack the transient outward potassium current (*I*_to_) (Hoppe et al., 1999), which is critical for early phase 1 repolarization during the cardiac AP (Varro et al., 1993; Dixon et al., 1996). However, when Kv4.3 was artificially introduced and *I*_to_ could be recorded from the guinea pig myocytes, the guinea pig APD decreased dramatically (Hoppe et al., 1999). This is quite different from larger mammals, where *I*_to_ establishes the membrane potential for Ca^2+^ entry and influences APD. In addition, changes in *I*_to_ in larger animals may underlie ventricular tachycardia (Choi et al., 2018).

The *I*_to_ peak of 12.6 ± 1.2 pA/pF at +50 mV in freshly isolated 3wRbCMs (Figure 6F) is similar to our published *I*_to_ value of 12.4 ± 1.4 pA/pF in adult rabbit cardiomyocytes from the left ventricle (Choi et al., 2018), and *I*_to_ values of 11 and 14 pA/pF measured in sub-endocardial and sub-epicardial human left ventricle myocytes, respectively (Johnson et al., 2018). Of note, the main subunits that code for *I*_to_ in rabbits are Kv4.3 and Kv1.4 (Wang et al., 1999; Bosch et al., 2003; Rose et al., 2005). Kv1.4 is likely to underline the slow recovery kinetics of *I*_to_ in the rabbit (Wang et al., 1999). Therefore, we concentrated on the expression of these two genes which code for the majority of the fast and slow components of *I*_to_. Significantly, the ratio of the amplitudes of fast and slow inactivating components in 3wRbCMs (3.2) is similar to the ratio (2.9) found in adult rabbit cardiomyocytes from the left ventricle (Choi et al., 2018).

The amplitude of the *I*_Kr_ tail peak after the +30 mV depolarizing step was significantly larger in freshly isolated cells (1.1 ± 0.1 pA/pF) than in both non-transduced and GFP-transduced 3wRbCMs (Figure 7D). This implies a significant run-down of *I*_Kr_ during 48 h culturing, but no significant additional effect of GFP transduction. Yet, *I*_Kr_ amplitudes in both freshly isolated and cultured 3wRbCMs are still comparable to the *I*_Kr_ tail current in human cells (1 pA/pF) (Jost et al., 2005; O’hara et al., 2011) and cultured adult rabbit myocytes (0.8 pA/pF) (Roder et al., 2019). Hence, these cells form an excellent system to study the regulation of *I*_Kr_ by different modulators (Organ-Darling et al., 2013; Roder et al., 2019).

The *I*_Ks_ plateau current at the end of the +40 mV depolarizing step in freshly isolated 3wRbCMs was fairly small, 0.43 ± 0.07 pA/pF (Figure 8), which is about two times smaller than in adult rabbit ventricular cardiomyocytes (Bartos et al., 2017). However, *I*_Ks_ was undetectable in 3wRbCMs after 48 h of culturing. Therefore, *I*_Ks_ contributes less to AP repolarization relatively to *I*_Kr_ in freshly isolated 3wRbCMs and do not contribute to AP repolarization in the cultured cells. Cumulative data on the amplitudes of studied ion currents and corresponding channel protein expressions can be found in Supplementary Tables 1, 2.

Sex hormones affect rabbit arrhythmogenesis, for example in long-QT-syndrome rabbits (Odening and Koren, 2014). However, an infantile phase of development in rabbits extends from birth to 40 days, characterized by low testosterone and follicle stimulating hormone levels, along with the slow growth of testis and seminal vesicle, while the peripubertal phase starts abruptly around day 40 (Berger et al., 1982). For example, the relative testicular weight of male rabbits from birth to 30 days was less than 3 mg per 100 g of body weight, while at the age 210 days the relative testicular weight increased to about 180 mg per 100 g of body weight, i.e., 60 times increase per 100 g of body weight. Since all rabbits in our model are 21–27 days old, which is almost twice younger than the beginning of the peripubertal phase, we neither expected nor noticed any significant difference between male and female 3wRbCMs. Therefore, we randomly used both male and female 3-week-old rabbits and measured the average values of the studied parameters (Moshal et al., 2019; Turan et al., 2020).

### Specific and Impartial Limitations of Cultured 3-Week-Old Ventricular Cardiomyocytes

The major limitation of the 3wRbCM system is that during 48 h of culturing the cells undergo significant electrical remodeling, which leads to significant changes in channel expression and corresponding ionic currents and APD. We found that total expression of all examined channel proteins and the electrical membrane capacitance were significantly decreased during 48 h in culture in both non-transduced and GFP-transduced cells. This decrease correlates with the reduction of most of the corresponding currents normalized to the capacitance. However, the electrical remodeling is a problem inherent to all models of cultured primary cardiomyocytes (Mitcheson et al., 1996, 1997; Burstein et al., 2007; Uesugi et al., 2014; St Clair et al., 2015; Li et al., 2017; Kopton et al., 2020). Therefore, an investigation of the effects of exogenously expressed proteins in cultured 3wRbCMs should be carefully conducted with appropriate control. For example, to study the effect of a gene of interest, X, cells should be transduced with a respective viral vector expressing X and a reporter gene such as GFP. As a control, cells from the same preparation would be transduced with a vector expressing GFP only. Patch clamp recordings would then be performed within the same time frame switching between cells from both groups.

## Conclusion

In summary, freshly isolated 3wRbCMs have all major channels necessary to generate the AP and can be infected with adenovirus encoding molecules of interest and cultured for 48h. Infected with a protein of interest and cultured for 48 h, 3wRbCMs preserve the structure and function of most of the channels. Therefore, most currents reported in this study (*I*_Na_, *I*_Ca,L_, *I*_K1_, *I*_to_, and *I*_Kr_) can be measured and further investigated after 48 h in culture. However, *I*_Ks_, runs down after 48 h of culturing to an undetectable level, and therefore cannot be investigated in a 3wRbCMs culture. Thus, we conclude that the 3wRbCMs model is a valuable, low-cost alternative to adult rabbit cardiomyocytes to study molecular mechanisms of human-like cardiac excitation.

## Data Availability Statement

The original contributions presented in the study are included in the article/Supplementary Material, further inquiries can be directed to the corresponding author.

## Ethics Statement

The animal study was reviewed and approved by the Rhode Island Hospital Institutional Animal Care and Use Committee.

## Author Contributions

AK designed and performed most of the electrophysiological experiments, analyzed the electrophysiological and calcium transient data, prepared the figures, and wrote the manuscript. ES took confocal images and participated in electron microscopy imaging. YL established and isolated ventricular myocytes, performed T-tube and α-actinin staining, and KvLQT1 western blot analysis. KR created the GFP-expressing adenovirus, performed western blot analysis, and helped with manuscript writing. PB conducted electrophysiological recordings and Ca^2+^ transient measurements. BB conducted Ca^2+^ transient measurements. NT and KM generated pilot western blot figures. GK supervised the project, reviewed the data, and wrote the manuscript. All authors contributed to the article and approved the submitted version.

## Conflict of Interest

The authors declare that the research was conducted in the absence of any commercial or financial relationships that could be construed as a potential conflict of interest.

## Publisher’s Note

All claims expressed in this article are solely those of the authors and do not necessarily represent those of their affiliated organizations, or those of the publisher, the editors and the reviewers. Any product that may be evaluated in this article, or claim that may be made by its manufacturer, is not guaranteed or endorsed by the publisher.
