# Corrigendum: Three-week-old rabbit ventricular cardiomyocytes as a novel system to study cardiac excitation and EC coupling

**DOI:** 10.3389/fphys.2023.1157712

**Published:** 2023-02-22

**Authors:** Anatoli Y. Kabakov, Elif Sengun, Yichun Lu, Karim Roder, Peter Bronk, Brett Baggett, Nilüfer N. Turan, Karni S. Moshal, Gideon Koren

**Affiliations:** ^1^ Department of Medicine, Division of Cardiology, Cardiovascular Research Center, Rhode Island Hospital, The Warren Alpert Medical School of Brown University, Providence, RI, United States; ^2^ Department of Pharmacology, Institute of Graduate Studies in Health Sciences, Istanbul University, Istanbul, Türkiye

**Keywords:** cardiac ventricular myocytes, cultured, rabbit, EC coupling, patch clamp, cardiac excitation, drug discovery

In the published article, there was an error regarding the affiliations for Elif Sengun. As well as having affiliation 1, she should also be affiliated with “Department of Pharmacology, Institute of Graduate Studies in Health Sciences, Istanbul University, Istanbul, Türkiye.”

The authors apologize for this error and state that this does not change the scientific conclusions of the article in any way. The original article has been updated.

