# Supplementary figures and images for: Three-Week-Old Rabbit Ventricular Cardiomyocytes as a Novel System to Study Cardiac Excitation and EC Coupling
Source: Front Physiol. 2021 Nov 18;12:672360. doi: 10.3389/fphys.2021.672360 (PMC8637404; doi:10.3389/fphys.2021.672360)

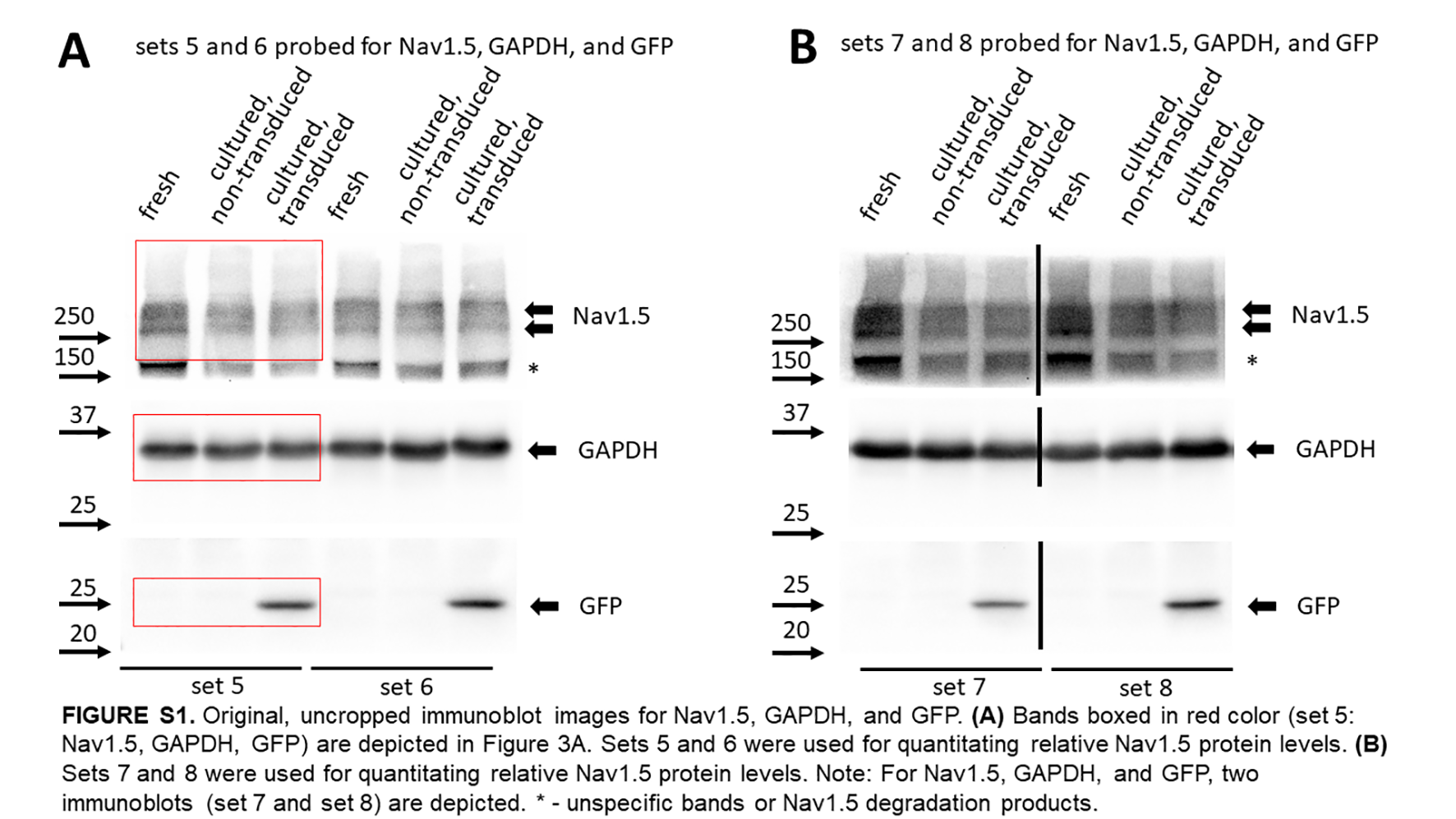

Supplement: Supplementary file 1 [file Image_1.TIF]

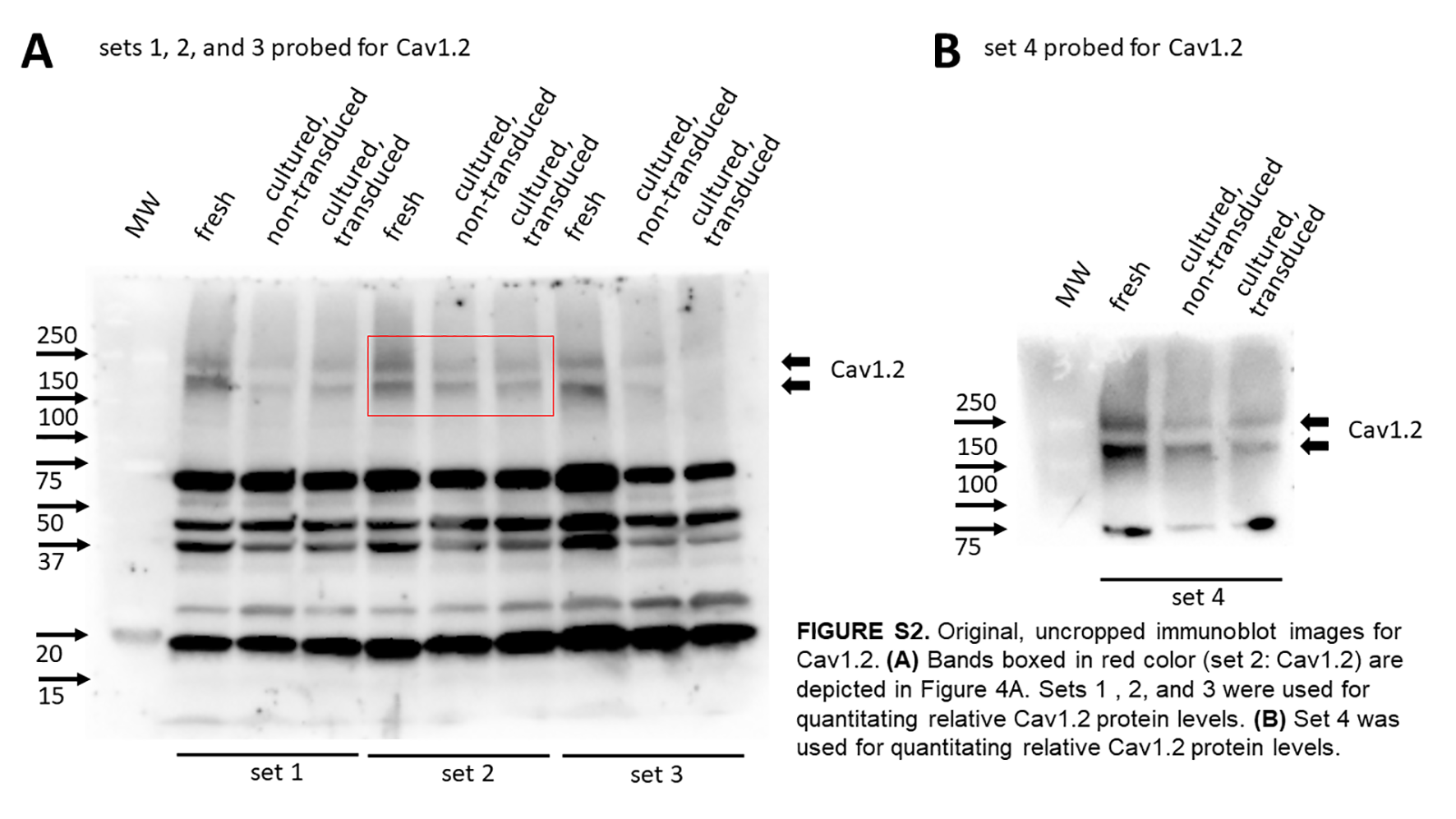

Supplement: Supplementary file 2 [file Image_2.TIF]

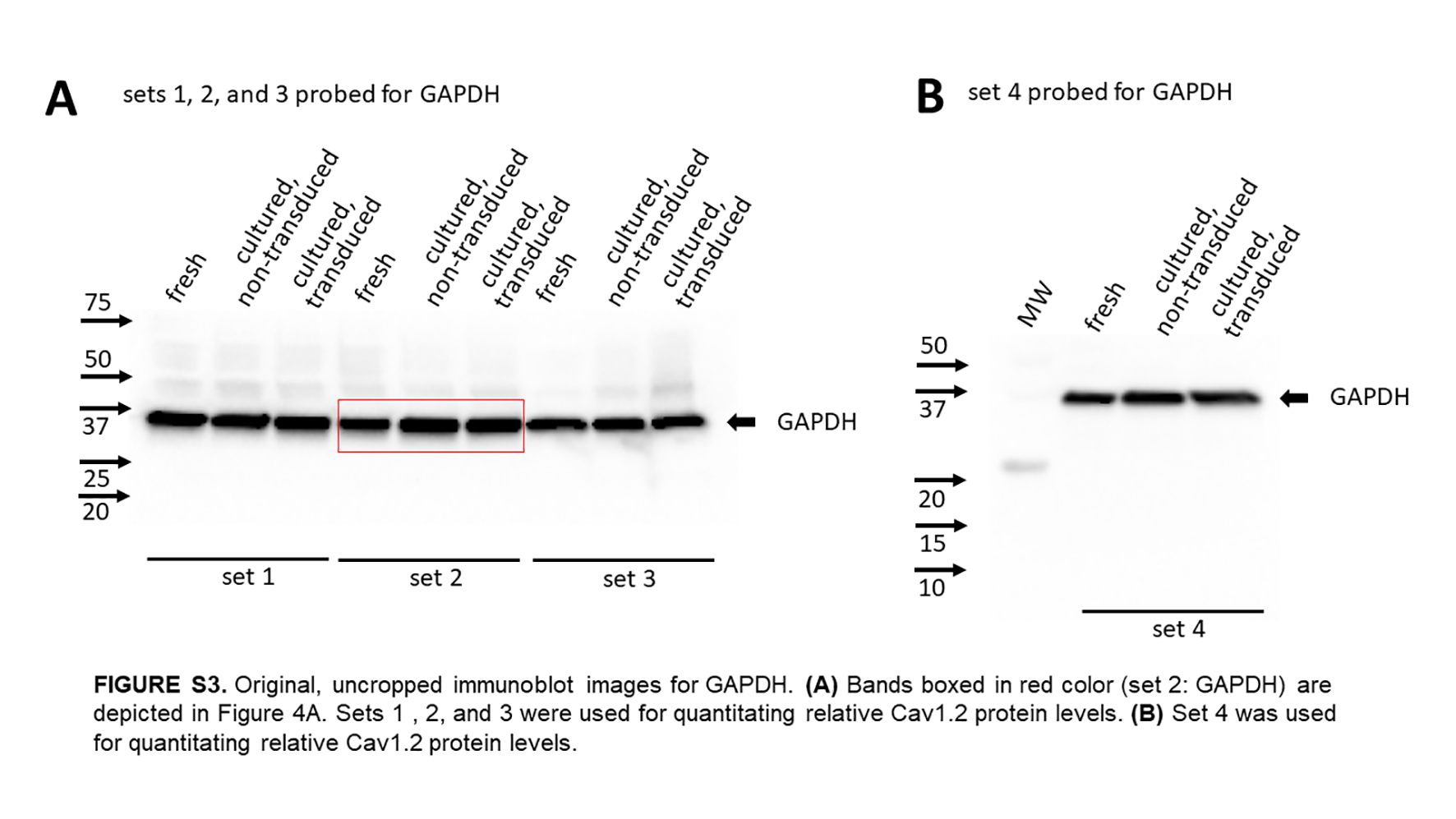

Supplement: Supplementary file 3 [file Image_3.TIF]

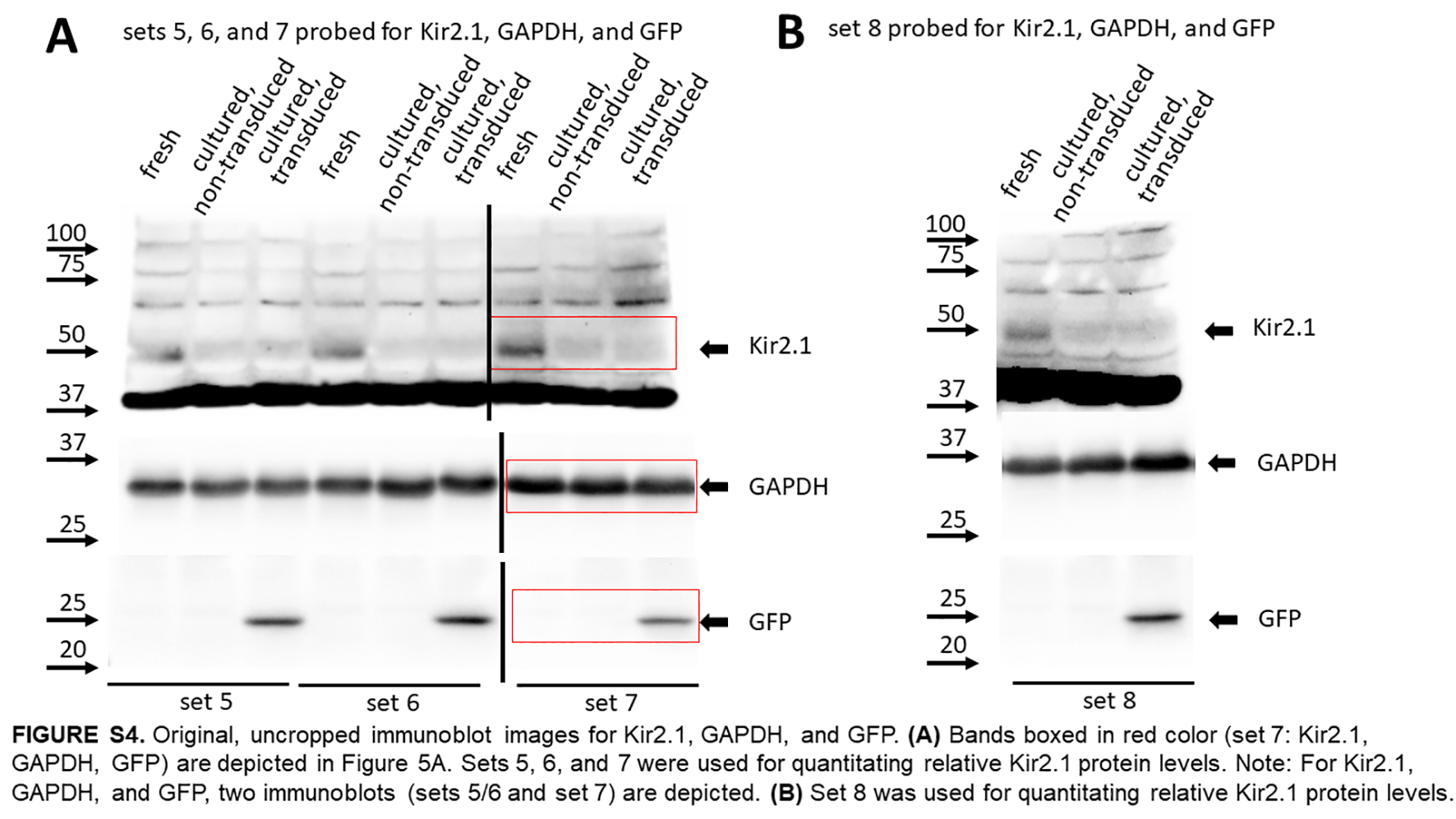

Supplement: Supplementary file 4 [file Image_4.TIF]

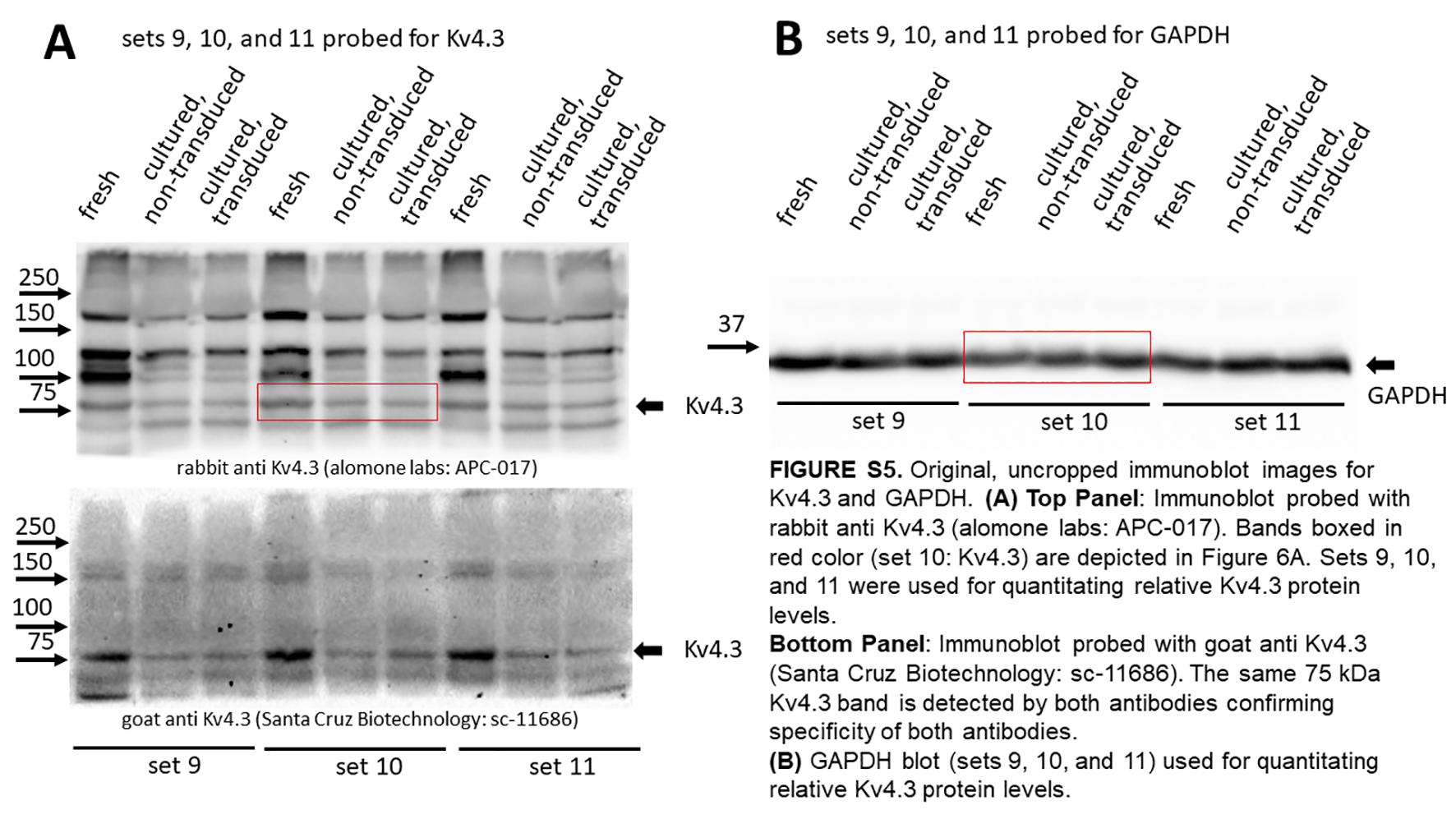

Supplement: Supplementary file 5 [file Image_5.TIF]

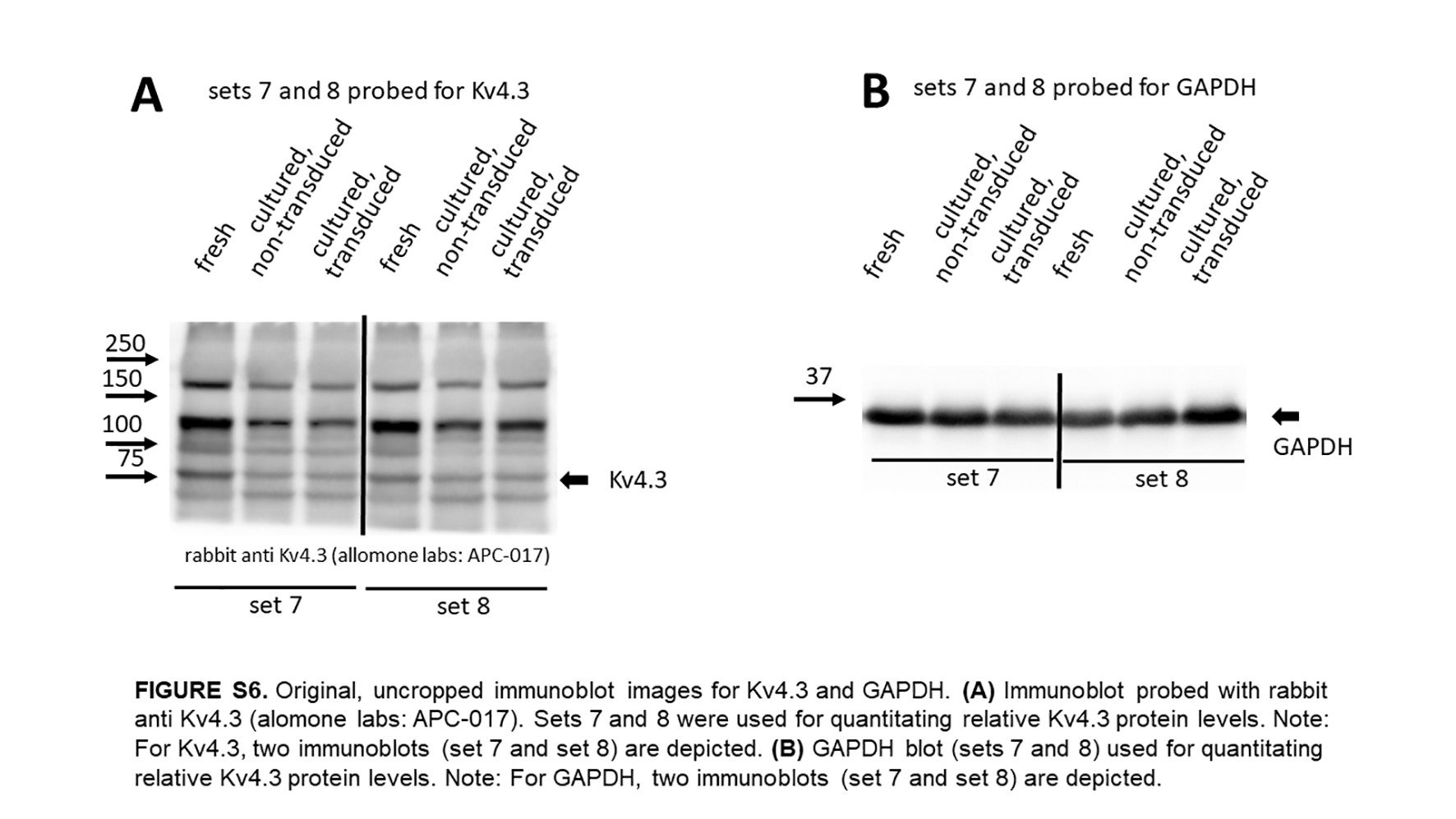

Supplement: Supplementary file 6 [file Image_6.TIF]

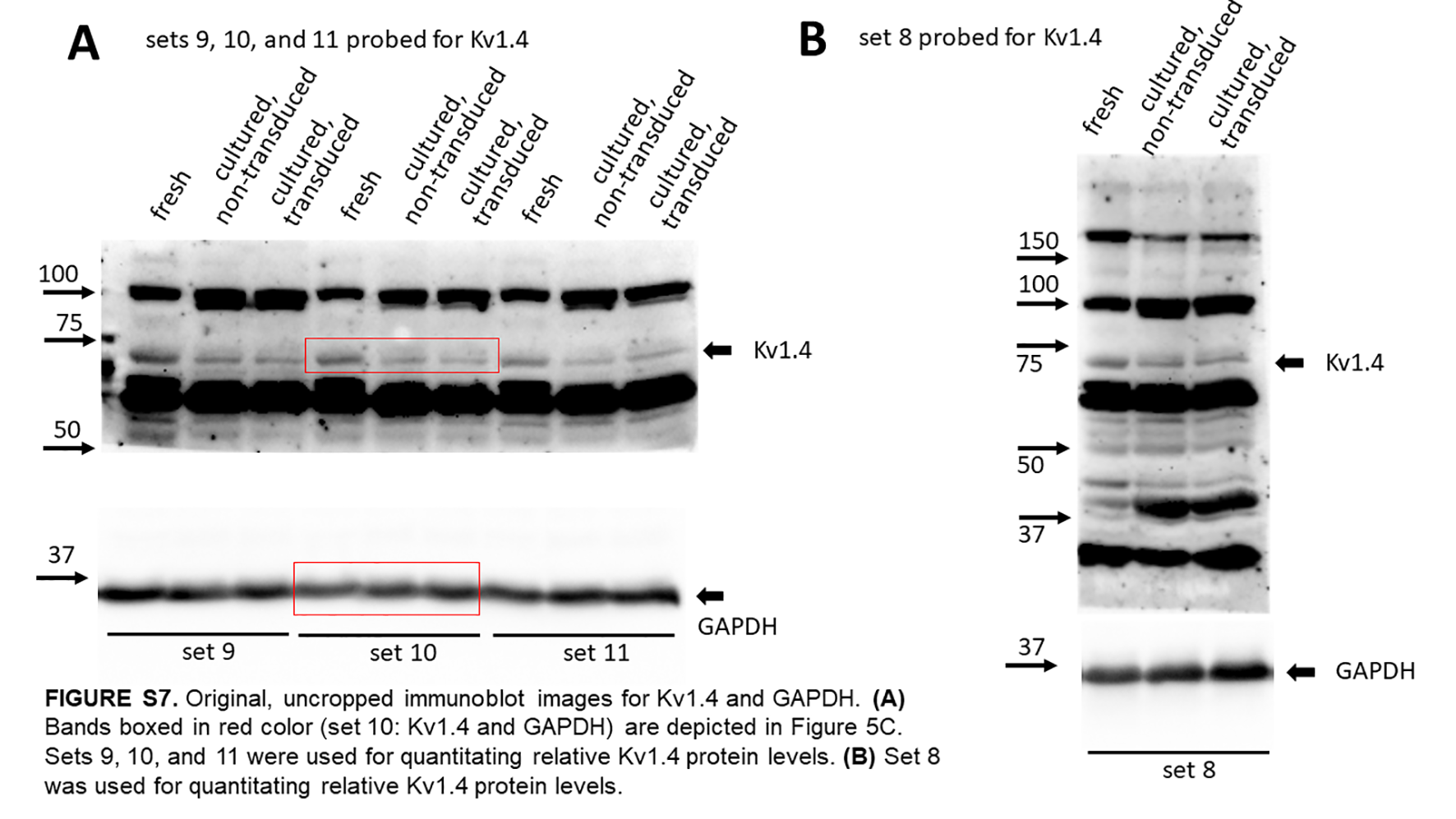

Supplement: Supplementary file 7 [file Image_7.TIF]

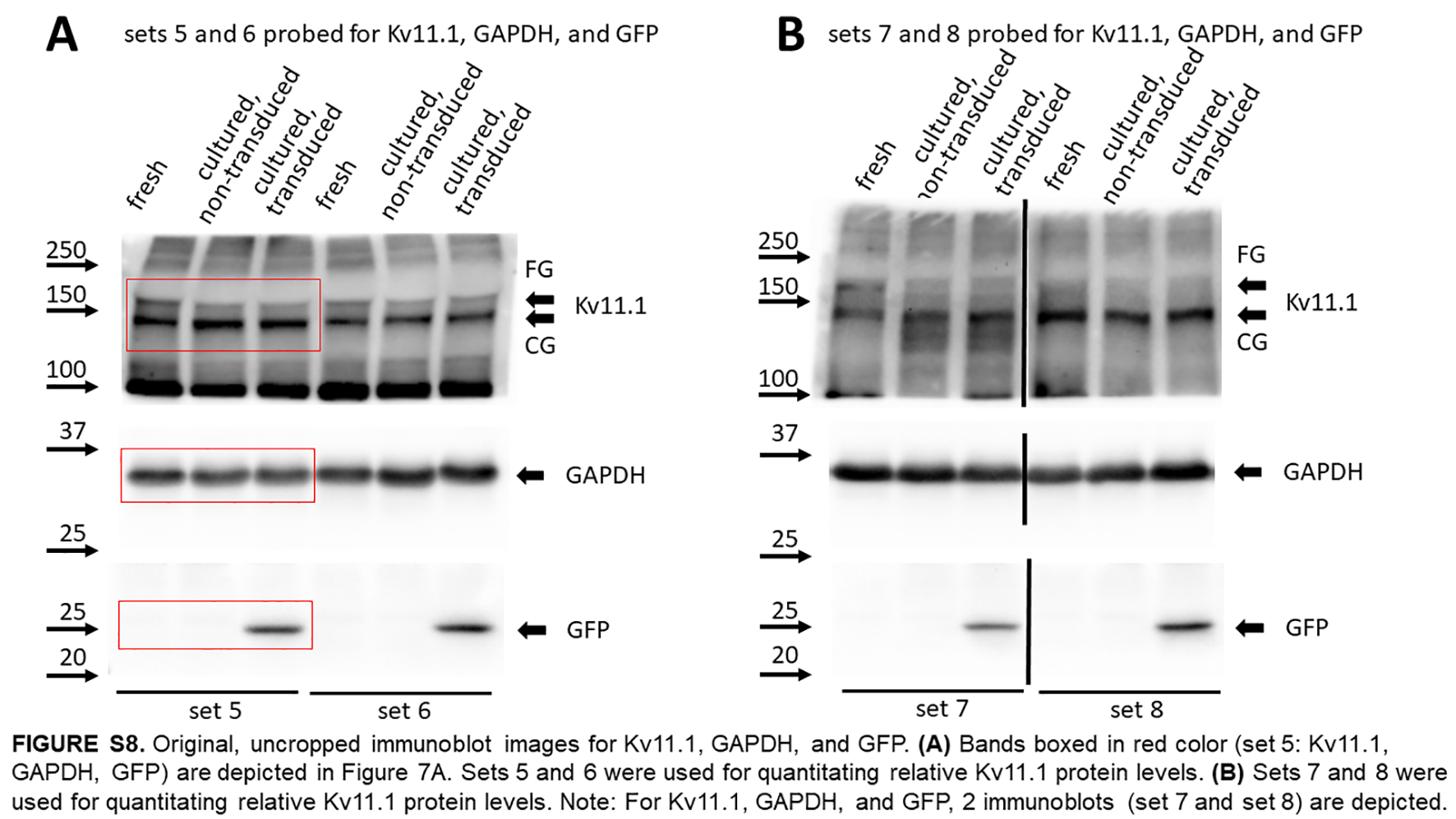

Supplement: Supplementary file 8 [file Image_8.TIF]

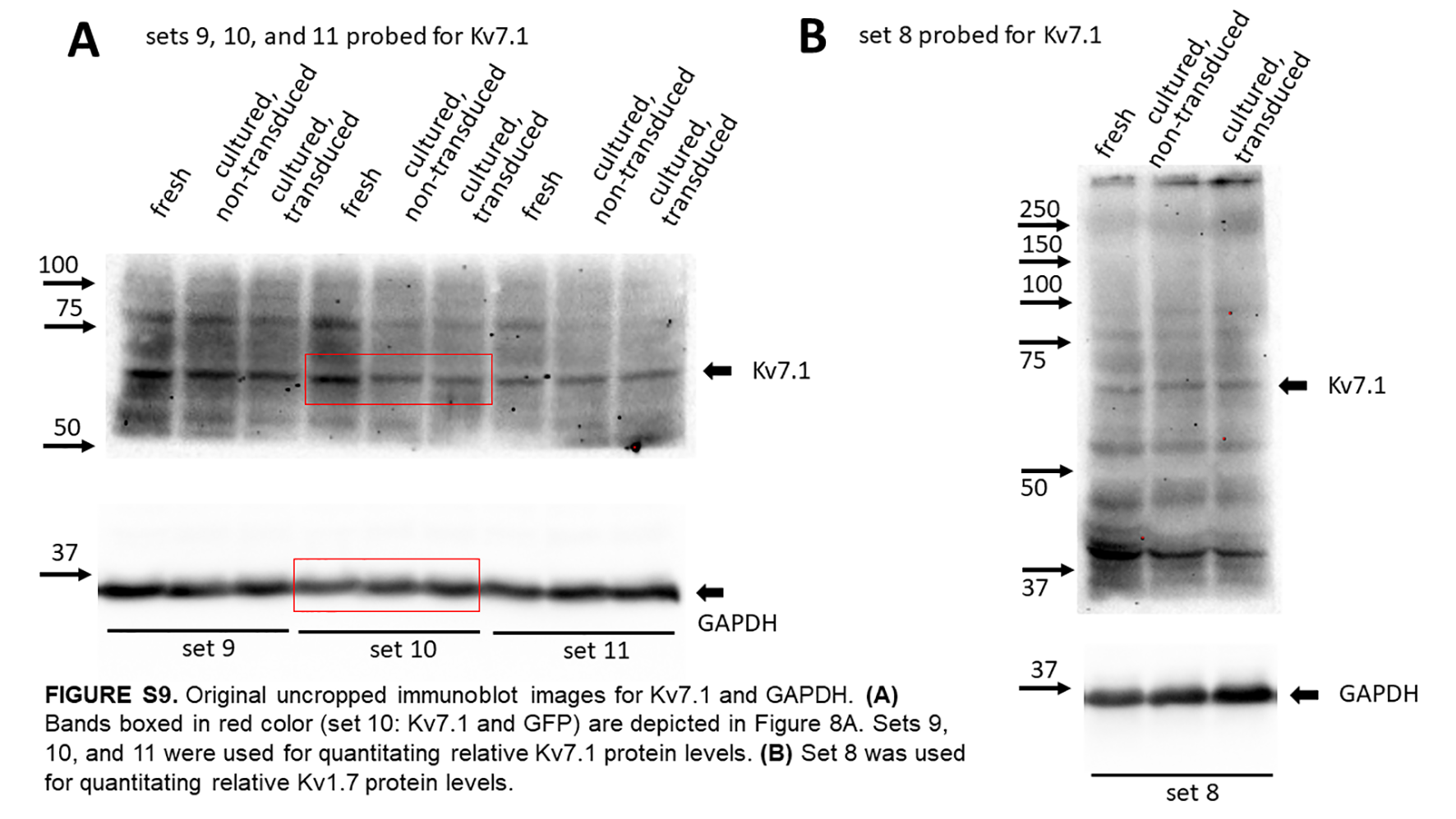

Supplement: Supplementary file 9 [file Image_9.TIF]

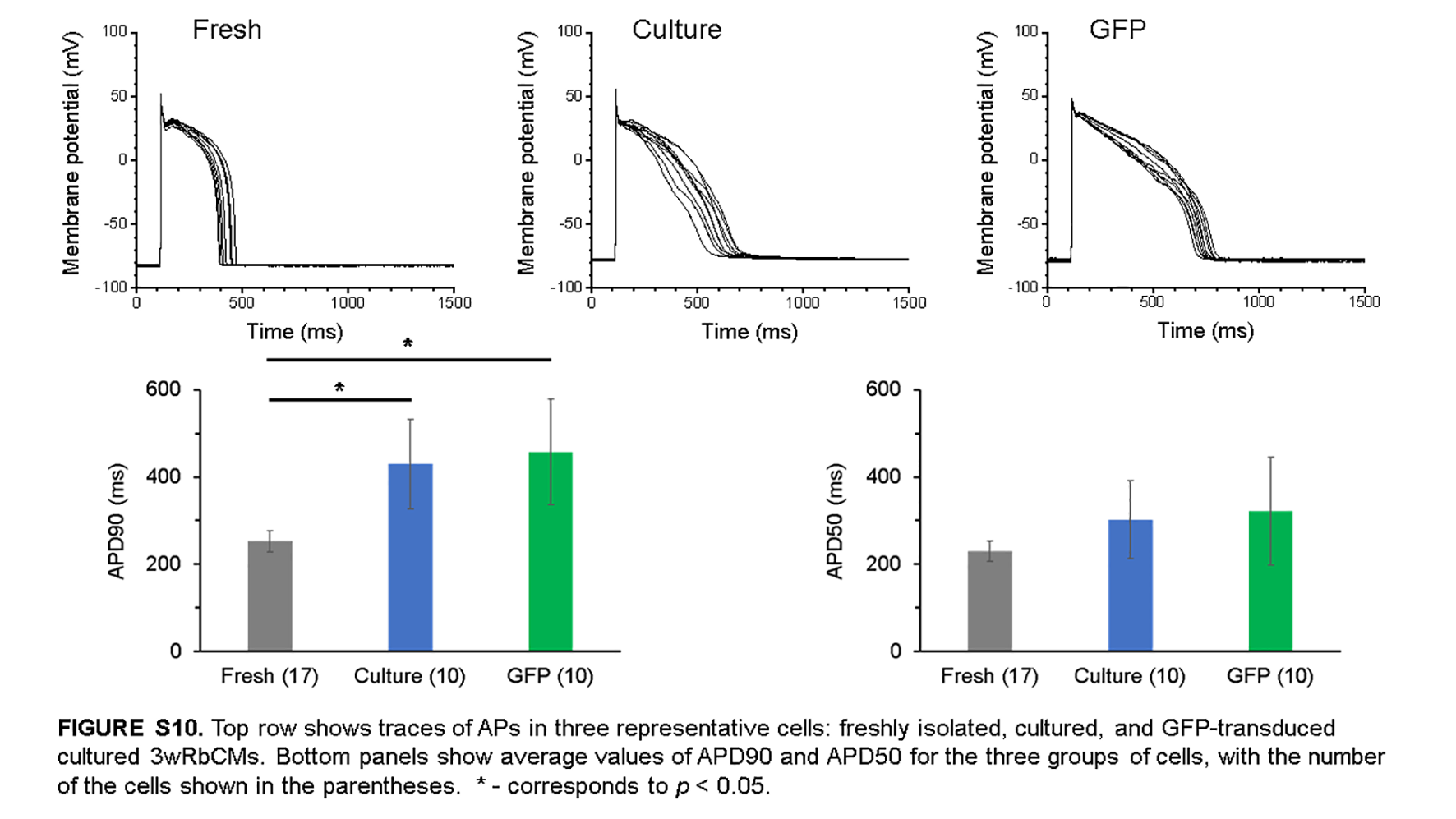

Supplement: Supplementary file 10 [file Image_10.TIF]

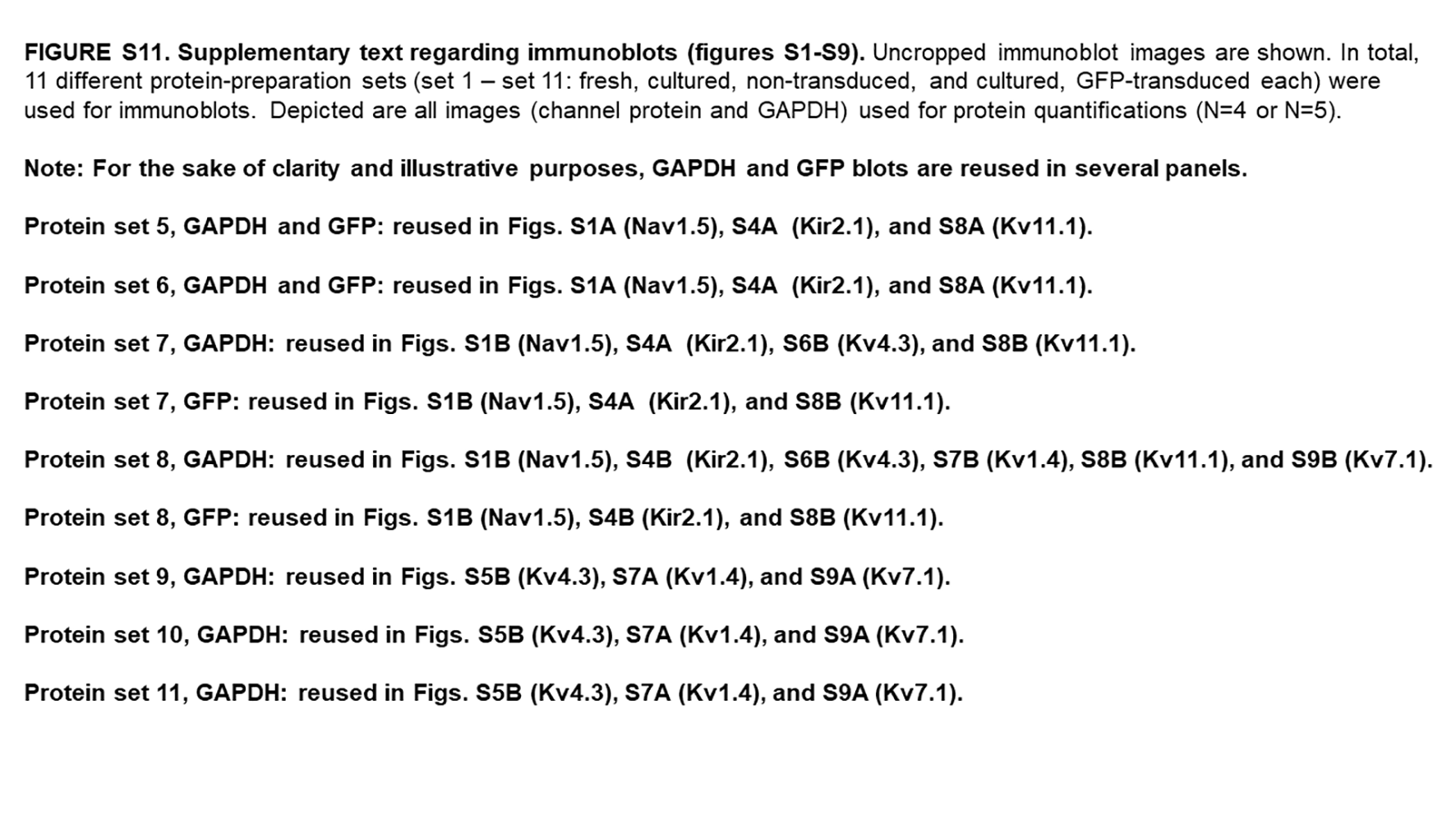

Supplement: Supplementary file 11 [file Image_11.TIF]
