# Supplementary material for: Three-Week-Old Rabbit Ventricular Cardiomyocytes as a Novel System to Study Cardiac Excitation and EC Coupling
Source: Front Physiol. 2021 Nov 18;12:672360. doi: 10.3389/fphys.2021.672360 (PMC8637404; doi:10.3389/fphys.2021.672360)
Supplement: Supplementary file 12 [file Table_1.pdf]

**Current densities (pA/pF)**				**Normalized total channel expression**			
**Current**	**Fresh**	**Culture**	**GFP**	**Protein**	**Fresh**	**Culture**	**GFP**
I_{Na} (-20 mV)	-158 ± 42	-135 ± 43	-32 ± 9 **	Nav1.5	1.00 ± 0.06	0.76 ± 0.06*	0.70 ± 0.08*
$I_{Ca,L}$ (0 mV)	-6.5 ± 0.5	-8.7 ± 0.7 *	-6.8 ± 0.7	Cav1.2	1.0 ± 0.3	0.21 ± 0.06**	0.23 ± 0.06**
I_{K1} (-120 mV)	-106 ± 2	-31 ± 5 **	-20 ± 2 **	Kir2.1	1.0 ± 0.3	0.33 ± 0.04**	0.36 ± 0.05**
I_{to} (+60 mV)	13.6 ± 1.2	12.2 ± 2.2	9.6 ± 0.8*	Kv4.3	1.0 ± 0.2	0.47 ± 0.04**	0.49 ± 0.05**
				Kv1.4	1.00 ± 0.10	0.38 ± 0.15**	0.39 ± 0.17**
I_{Kr} (+40 mV)	1.2 ± 0.1	0.47 ± 0.04**	0.69 ± 0.08**	Kv11.1 FG	1.0 ± 0.3	0.43 ± 0.11**	0.46 ± 0.13**
				Kv11.1 CG	1.0 ± 0.4	1.2 ± 0.4	1.2 ± 0.5
I_{Ks} (+40 mV)	0.38 ± 0.08	Not detected	Not detected	Kv7.1	1.0 ± 0.06	0.89 ± 0.13	0.76 ± 0.06 *

Table S1: Summary of current densities (mean ± SEM) and respective total channel protein levels (mean ± SD) in acutely isolated, cultured, and cultured, GFP-transduced 3wRbCMs. * and ** - correspond to $p < 0.05$ and $p < 0.01$, respectively.
