# Supplementary material for: Three-Week-Old Rabbit Ventricular Cardiomyocytes as a Novel System to Study Cardiac Excitation and EC Coupling
Source: Front Physiol. 2021 Nov 18;12:672360. doi: 10.3389/fphys.2021.672360 (PMC8637404; doi:10.3389/fphys.2021.672360)
Supplement: Supplementary file 13 [file Table_2.pdf]

		Fresh	Culture	GFP
**APD90** (ms)		253 ± 24	430 ± 103 *	457 ± 121 *
**APD50** (ms)		230 ± 24	302 ± 89	321 ± 123
I_{Na}	$g_{Na,max}$ (nS/pF)	3.0 ± 0.3	2.9 ± 0.3	0.8 ± 0.1 **
	$V_{1/2,Na}$ (mV)	-33 ± 2	-31 ± 1	-29 ± 2
	$V_{z,Na}$ (mV)	5.2 ± 0.5	5.0 ± 0.3	7.0 ± 0.6
$I_{Ca,L}$	$g_{Ca,max}$ (nS/pF)	161 ± 16	237 ± 20 *	178 ± 11
	$V_{1/2,Ca}$ (mV)	-11 ± 2	-14 ± 1	-14 ± 1
	$V_{z,Ca}$ (mV)	5.9 ± 0.6	6.7 ± 0.7	5.9 ± 0.4
I_{to}	τ_{fast} (ms)	7 ± 1		
	τ_{slow} (ms)	117 ± 28		

Table 2: Summary of measurements for APD values and selected ion channels kinetic parameters in acutely isolated, cultured, and GFP-transduced cultured 3wRbCMs (mean ± SEM). * and ** - correspond to $p < 0.05$ and $p < 0.01$, respectively.
